# Individualized prediction of clinical progression to dementia using plasma biomarkers in non-demented elderly

**DOI:** 10.1186/s13195-025-01925-1

**Published:** 2025-12-03

**Authors:** Madison I. J. Honey, Ingrid S. van Maurik, Argonde C. van Harten, Mariam Gouda, Mardou van Leeuwenstijn, Arenda Mank, Calvin Trieu, Vincent Bouteloup, Geneviève Chêne, Isabelle Pellegrin, Carole Dufouil, James D. Doecke, Christopher J. Fowler, Colin L. Masters, Yolande Pijnenburg, David Wilson, Wiesje M. van der Flier, Charlotte E. Teunissen, Inge M. W. Verberk

**Affiliations:** 1 Department of Laboratory Medicine, Neurochemistry Laboratory, Amsterdam University Medical Centers, Vrije Universiteit, Amsterdam Neuroscience, Amsterdam, The Netherlands; 2 Department of Epidemiology and Data Science, Amsterdam University Medical Centers, Vrije Universiteit, Amsterdam, The Netherlands; 3 Northwest Academy, Northwest Clinics Alkmaar, Alkmaar, The Netherlands; 4 Department of Neurology, Alzheimer Center Amsterdam, Amsterdam University Medical Centers, Vrije Universiteit, Amsterdam Neuroscience, Amsterdam, The Netherlands; 5 Inserm UMR1219 and CIC 1401 EC, Pôle Santé Publique, University of Bordeaux, Bordeaux Population Health, Centre Hospitalier Universitaire (CHU) de Bordeaux, Bordeaux, France; 6 CSIRO Health and Biosecurity/Australian E-Health Research Centre, Herston, QLD Australia; 7 The Florey Institute, The University of Melbourne, Parkville, VIC Australia; 8 Quanterix Corporation, Billerica, MA USA

**Keywords:** Blood, Plasma, Biomarkers, Prognosis, Dementia, Alzheimer’s disease, Risk, Individualised, Mild cognitive impairment, Subjective cognitive decline

## Abstract

**Background:**

We aimed to develop individualized predictions for risk of developing any-cause dementia and Alzheimer’s disease (AD) dementia, in individuals with subjective cognitive decline (SCD) or mild cognitive impairment (MCI), using plasma phosphorylated-tau-181 (pTau181), phosphorylated-tau-217 (pTau217; in a subset), amyloid beta1-42/1–40 (Aβ42/40), glial fibrillary acidic protein (GFAP) and/or neurofilament light (NfL).

**Methods:**

From the Amsterdam Dementia Cohort we included 314 individuals with SCD (age 61 ± 9 years, *n* = 184 (59%) male, MMSE 29 ± 1) and 253 individuals with MCI (age 65 ± 7 years, *n* = 165 (65%) male, MMSE 27 ± 2), who had annual follow-up (median duration 2.4 years). Cox proportional hazards regression models were used to calculate probabilities for progression to dementia and were externally validated in MEMENTO and AIBL cohorts.

**Results:**

During follow-up 20 SCD and 99 MCI patients developed dementia. For MCI patients who progressed to any form of dementia, plasma GFAP contributed on top of age, sex, and MMSE score in the parsimonious individualized prognostic model (C-index = 0.69 [95%CI = 0.63; 0.76]). With AD-dementia as the outcome, GFAP and pTau181 were selected in the parsimonious model on top of the demographic variables (C-index = 0.71 [95%CI = 0.65; 0.76]). In the subset of 197 MCI individuals with pTau217 measurements, pTau217 was selected in the parsimonious model on top of the demographic variables (C-index = 0.75 [95%CI = 0.69; 0.79]). External validation demonstrated that the models are robust in a memory clinic setting.

**Conclusions:**

Our prediction models have utility for clinical practice to calculate progression probabilities for development of dementia in individual patients living with MCI over a 1-, 3- and 5-year time period.

**Supplementary Information:**

The online version contains supplementary material available at 10.1186/s13195-025-01925-1.

## Background

Individuals with subjective cognitive decline (SCD) or mild cognitive impairment (MCI) experience a decline in their cognitive abilities but have not yet met the criteria for a diagnosis of dementia. SCD refers to a self-perceived decline in cognition in the absence of objective impairment on standardized cognitive tests. In contrast, MCI is defined by objective evidence of cognitive impairment on neuropsychological testing, without instrumental interference of daily functioning [1]. Individuals with SCD or MCI are at a higher risk to develop dementia compared to individuals without cognitive complaints [2]. The risk of progression increases if there is underlying AD pathology, which can be measured using cerebrospinal fluid (CSF) biomarkers or positron emission tomography (PET), and it has been previously demonstrated that these biomarkers can be utilized as prognostic tools to aid in individualised risk prediction [3, 4]. These biomarker-based prognostic models have primarily been designed for personalized risk calculation in MCI populations, with limited application for individuals with SCD [5]. However, even at the MCI stage, there is reluctance to use these biomarkers, in part due to their perceived invasiveness, high costs and/or requirement for specialized equipment [6].

Blood-based biomarkers represent a less invasive and less expensive alternative, with the possibility to simultaneously measure multiple biomarkers from one sample to capture different aspects of disease pathology, alike for CSF [7, 8]. Blood-based biomarkers could be used within the near-future diagnostic work-up of memory clinic patients with cognitive complaints, as stand-alone diagnostic tests for AD pathology since their accuracy appears to be equivalent to CSF tests for detection of abnormal amyloid PET in the intended-use population (a sensitivity and specificity of ~ 90%) [9, 10]. Key blood biomarkers for dementia include phosphorylated-tau-217, phosphorylated-tau-181, amyloid beta 1–42/1–40, glial fibrillary acidic protein and neurofilament light [11]. Former studies have shown that these blood-based biomarkers can be used to inform about the risk of progression to dementia at a group level [12–19], however, their demonstrated utility for individualized prognosis remains extremely limited [18].

Patients express a desire to be informed of their potential clinical trajectory, and given that 30–40% of dementia cases are caused by dementias other than AD [20, 21], progression to any form of dementia is a highly relevant outcome for individuals with MCI [22–24]. Using individualized prediction models to identify which individuals with SCD or MCI are at a higher risk of progressing to dementia may help guide clinical decision making and enable high-quality individualized care, as prevention strategies can be different depending on individuals with different risks of dementia [25–27].

We therefore aimed to develop and validate prediction models utilizing blood-based biomarkers pTau181, pTau217, Aβ42/40, GFAP and/or NfL, for individualized determination of any-cause dementia risk as well as specifically Alzheimer’s dementia risk in a memory clinic sample of patients with MCI, or SCD.

## Methods

### Participants

The cohort used for model development was comprised of individuals from the Amsterdam Dementia Cohort [28] and SCIENCe project [29], who visited the tertiary memory clinic at the Alzheimer Center Amsterdam (Vrije Universiteit Amsterdam, Amsterdam UMC location VUmc, Amsterdam, Netherlands) for diagnostic purposes between 2004 and 2019. At baseline, individuals had an initial clinical evaluation with a physician and underwent diagnostic workup including physical, neurological and neuropsychological evaluations, brain MRI, apolipoprotein E (APOE) genotyping and CSF AD biomarker analysis (Aβ42, pTau181, total-tau). Individuals were then diagnosed by a multidisciplinary team. They were classified as having subjective cognitive decline (SCD) when they presented to the clinic with cognitive complaints but had no objective impairment as measured on their neuropsychological and neurological evaluations, and did not meet criteria for other neurological or psychiatric disorders. A diagnosis of mild cognitive impairment (MCI) was given based on clinical criteria which includes a deficit in one cognitive domain without instrumental interference of daily living activities [1]. Individuals were included in the study if they had at least one follow-up diagnosis with a minimum of six months between the baseline and follow-up visit and a plasma sample collected within six months of their initial diagnosis. These individuals were a subsample from a previously published study [30].

### Clinical follow-up

Individuals returned for annual follow-up visits, during which neurological and neuropsychological evaluations were repeated and a diagnosis was given accordingly following each visit during a multidisciplinary consensus meeting. Diagnoses were made in accordance with research criteria for Alzheimer’s Disease dementia [31], vascular dementia [32], dementia with Lewy bodies [33], frontotemporal dementia [34], and progressive supranuclear palsy [35]. We used clinical progression to any-cause dementia from SCD or MCI at baseline as the outcome measure. For secondary analysis, we used clinical progression to specifically AD dementia as the outcome measure. The time to progression was defined as the time between the date of plasma sampling and the date at which the diagnosis first changed to any or AD dementia. Clinical diagnoses at follow-up were established independently of baseline plasma biomarker results, ensuring blinding of outcome assessment.

### Biomarker analyses

GFAP, NfL, Aβ40 and Aβ42 (for all analyses we used the Aβ42/Aβ40 ratio) were measured in EDTA plasma samples (one aliquot per sample) as previously described [30]. In a subset of individuals, pTau217 was measured in duplicate from a different, second aliquot of the same sample compared to all other biomarkers, using the Simoa ACC (Janssen) pTau217 assay kit (Quanterix; Kit Lot MW20231107), on the Simoa HD-X analyzer. All biomarkers were measured blinded to participants’ clinical information and follow-up status. pTau217 measurements were only available for a subset of the study population, for which too few individuals progressed from SCD, therefore clinical progression to any-cause dementia from MCI at baseline was used as the only outcome measure.

### Model external validation in MEMENTO and AIBL cohorts

We performed external validation of our models on a sample comprising individuals with MCI from the MEMENTO study and the Australian Imaging, Biomarker & Lifestyle (AIBL) study.

The MEMENTO cohort comprises individuals with cognitive complaints who visited one of twenty-six memory clinics within the French national network of university-based memory clinics, between 2011 and 2014. For validation purposes, the baseline MEMENTO sample included individuals presenting with MCI (defined by a clinical dementia rating (CDR) score of 0.5) at 2-years visit after their enrollment to the MEMENTO study (all the blood biomarkers were available at this time point). These individuals were followed at clinical sites yearly, until 5 years after enrollment (3 years after baseline.) Incident dementia cases were reviewed by an expert panel following international criteria [36].

AIBL is an observational longitudinal cohort study where participants were recruited on a voluntary basis from the general population in Australia from 2006 onwards. Individuals received a diagnosis of MCI according to international criteria [1, 37]. Participants underwent reassessment every 18 months and were reviewed by a clinical panel including old age psychiatrists, a neurologist, a geriatrician, and neuropsychologists, whereby diagnostic classifications were given based on international criteria [38]. For both AIBL and MEMENTO cohorts, clinical diagnoses at follow-up were established independently of baseline plasma biomarker results.

GFAP was measured using Simoa N4PE kits in both cohorts, pTau181 was measured with pTau-181 Advantage V2.1 assay kits in both cohorts, and pTau217 was additionally measured in AIBL using Simoa ACC pTau217 assay kits, all according to manufacturer’s instructions. In AIBL, biomarkers were measured in EDTA plasma samples, in MEMENTO, GFAP was measured in EDTA plasma whereas pTau181 was measured in serum samples [39]. All biomarkers were measured blinded to participants’ clinical information and follow-up status.

For the analysis, biomarker results were standardized as Z-scores to allow comparisons across the three studies. The previously established parsimonious models and the model regression coefficients for both progression to any-cause dementia and progression to AD dementia were applied to the validation data. The parsimonious model utilising pTau217 for progression to any-cause dementia was additionally externally validated in AIBL. The Harrell’s C-statistic was calculated to assess model discrimination in the validation cohorts. Model calibration was assessed by superimposing observed and expected survival as predicted by the models in the validation cohorts. For this, we categorized the prognostic index of the models, that is, the sum of the regression coefficients multiplied by the value of their respective predictors, at the 16th, 50th and 84th percentiles to define four risk groups; good prognosis (> 84th percentile), fairly good prognosis (50–84th percentile), fairly poor prognosis (16–50th percentile), and poor prognosis (< 16th percentile). Classification of the prognostic index in these groups is in line with consensus guidelines for external validation of cox proportional hazard models, with the cut-points corresponding to 0 and approximately ± 1, i.e. mean ± 1 standard deviation on a standard normal scale [40]. For external validation of the AD dementia model, the risk groups in all cohorts were defined by 25th, 50th and 75th percentiles due to the lack of incident cases in the 16th percentile in MEMENTO**.** Hazard ratios between these risk groups were compared between the ADC and validation cohorts.

### Statistical analysis

For data analysis and visualization we used R version 4.0.3. All plasma biomarkers were natural log-transformed (ln) and Z-scored (Z-scoring performed separately for the MCI and SCD population) before statistical analyses for comparability of effect sizes. A *p*-value < 0.10 was considered significant, to reduce the risk of excluding potentially relevant predictors too early during model development, in line with consensus guidelines for prediction modelling [41]. Sex was coded as 0 (male) or 1 (female). There were no missing data for the predictor variables (age, sex, MMSE score, pTau181, GFAP, NfL and Aβ42/Aβ40) or for the outcome variables; therefore, all analyses were performed using the complete dataset. As pTau217 results were available only for a subset of individuals with MCI, analyses including pTau217 were repeated in that subset, for which all other predictor and outcome variables were complete.

We constructed Kaplan–Meier survival plots with curves for low, middle, and high baseline biomarker concentrations (data in tertiles) to visualize the risk of clinical progression over time. We constructed prognostic models using Cox proportional hazards regression analysis, with clinical progression to any-cause dementia, and AD dementia, as two separate outcome measures. The time variable in our models was the time to progression to dementia, or the maximum duration for which an individual was followed if they had no clinical progression. Participants were followed until their last available clinical assessment and individuals who did not convert to dementia were censored at the date of their last known visit. When using AD dementia as the outcome measure, individuals who converted to another form of dementia were censored at the time of their conversion.

We first assessed a basic demographic-only model with age, sex and MMSE score to predict risk of incident dementia. We then expanded this model by including each plasma biomarker individually, and in addition combined all plasma biomarkers in one model to investigate the value of measuring a blood biomarker panel (all separate models). Finally, we constructed a parsimonious model using backward selection based on the Akaike information criterion (AIC), starting with the full model which included age, sex, MMSE score and all four biomarkers (pTau181, GFAP, NfL and Aβ42/Aβ40) and forcing the model to keep in the demographic variables during the backward selection. The resulting parsimonious model retained the fewest number of plasma biomarkers necessary to achieve satisfactory model fit. This approach was chosen to balance predictive accuracy with simplicity, thereby improving interpretability, reducing the risk of overfitting, and enhancing the potential generalizability of the model to external cohorts. For the subset of individuals with pTau217 results, the parsimonious model was constructed using backward selection based on the AIC starting with the full model which included age, sex, MMSE score and all five biomarkers (pTau217, pTau181, GFAP, NfL and Aβ42/Aβ40).

We assessed our prognostic models by their discrimination (Harrell’s C-index, a score closer to 1 indicates a better model) and accuracy (time-dependent Brier score, a score closer to 0 indicates a better model). The Brier score is used to quantify the accuracy of probabilistic predictions by comparing predicted probabilities to actual outcomes, providing a way to evaluate the reliability of individualized predictions. We calculated the c-index difference between each model and the demographic-only model (a positive difference indicates superior discriminative performance in the model of interest compared to the demographic-only model). Model parameters and 95% confidence intervals (95% CI) were estimated using bootstrapping to reduce the optimism in estimations. We used the parsimonious model to calculate 1, 3, and 5-year probabilities for progression to any-cause or AD dementia. Only for visualization purposes, we constructed risk charts with probabilities calculated for men and women, with a younger or older age (50 or 75 years old), an MMSE score ranging from 24 to 30 (a score below 24 is the most frequently used cut point to indicate dementia) [42] and with biomarkers utilised as categorical variables entered into the models (data in tertiles). However, the strength of our approach is that models can generate probabilities from continuous biomarker and demographic data, hence calculate probabilities for each level of the plasma biomarkers and the demographic variables. This was in addition visualized using the Shiny package in R: we developed an interactive interface where any combination of sex, age (range 50 to 75 years), MMSE score (range 24 to 30) and plasma biomarker concentration (GFAP range 14–299 pg/mL, pTau181 range 0.59–11.0 pg/mL and pTau217 range 0.006–0.500 pg/mL based on dataset range) can be entered to estimate the risk that an individual will develop any-cause or AD- dementia within one, three and five years. In this interface, probabilities are calculated on the demographic-only prognostic model, or on the parsimonious models if plasma biomarker data is entered.

Model development and validation were performed in accordance with the TRIPOD (Transparent Reporting of a multivariable prediction model for Individual Prognosis or Diagnosis) guidelines, and a completed TRIPOD checklist is provided in the Supplement. We performed all analyses separately for the individuals with SCD and the individuals with MCI, as well as for the MCI subset with pTau217 measurements. For the analyses in the SCD population, we were not able to assess models using all plasma biomarkers combined, or generate individualised predictions due to a too low number of individuals who converted to dementia during follow-up, i.e. the models did not converge.

## Results

### Model development and variable selection for risk of any-cause or AD dementia

From the 253 individuals with a baseline diagnosis of MCI in the ADC, 99 (39%) converted to dementia over a median (IQR) follow-up duration of 2.4 (1.2–3.6) years (Table 1). From the 314 individuals with SCD, 20 (6%) progressed to dementia during a median (IQR) follow-up duration of 4 (2.7–6.2) years (eTable 1).

**Table 1:** Baseline demographics and clinical characteristics of the MCI population, stratified for conversion to any-cause dementia at follow-up

	Total group	Stratified for diagnosis at follow-up	
		Stable	Progression to any-cause dementia
Number of participants	253 (100%)	154 (61%)	99 (39%)
Demographic and clinical characteristics			
Age, years	65 (7)	65 (7)	65 (7)
Sex			
Male	165 (65%)	109 (71%)	56 (57%)
Education (range 1–7)	5 (4–6)	5 (4–6)	5 (4–6)
Follow-up duration, years	2.4 (1.2–3.6)	2.2 (1.1–3.2)	3.0 (1.7–4.2)
Number of visits	3 (2—4)	3 (2—4)	3 (2—5)
Time to progression, years	–-	–-	2.8 (1.7–4.1)
Cognitive test performance			
MMSE	27 (25–28)	27 (26–28)	26 (25–28)
Plasma biomarkers			
NfL (pg/mL)	14.2 (11.0–19.0)	13.8 (10.7–17.9)	15.3 (11.4–20.1)
Aβ42/40	0.053 (0.048–0.059)	0.054 (0.048–0.061)	0.051 (0.047–0.056)
GFAP (pg/mL)	90.6 (67.2–129.6)	80.2 (57.8–109.6)	118.7 (88.0–141.7)
pTau181 (pg/mL)	1.86 (1.33–2.51)	1.57–1.18–2.15)	2.31 (1.69–2.83)

Data are n (%), mean (SD), or median (IQR) for the total group and stratified for individuals who received a diagnosis of dementia during follow-up or those who remained with a diagnosis of mild cognitive impairment at their last visit. The group who progressed to dementia during follow-up was comprised of 91 AD dementia, 3 Dementia with Lewy Bodies, 3 vascular dementia, 1 frontotemporal dementia and 1 mixed-cause dementia cases. Aβ42/40 = Amyloid β42/40. GFAP = glial fibrillary acidic protein. MMSE = mini-mental state examination. NfL = neurofilament light. pTau181 = phosphorylated-tau-181

**Fig. 1:**
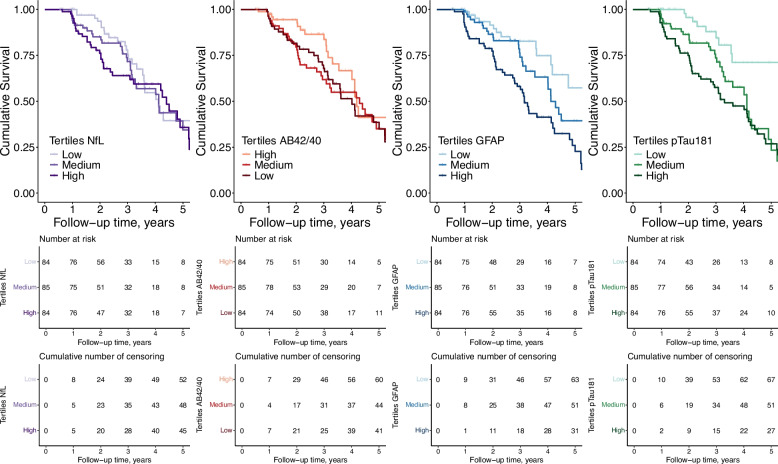
Kaplan–Meier curves of clinical progression to any-cause dementia from MCI for individuals with low, medium, or high baseline plasma biomarker levels. The Kaplan–Meier curves present the observed clinical progression to dementia over time, visualised for tertiles of baseline plasma biomarker concentrations. Curves are unadjusted for covariates age, sex and MMSE score. Aβ42/40 = Amyloid β42/40. GFAP = glial fibrillary acidic protein. NfL = neurofilament light. pTau181 = phosphorylated-tau-181

We first modelled the risk of progression to any-cause dementia in MCI patients. For this MCI sample, the demographics-only prognostic model including age, sex and mini-mental state examination (MMSE) score had a Harrell’s C-index of 0.62 (0.56–0.67; hazard ratios (HR) presented in Table 2). When adding the plasma biomarkers to the demographics-only model (separate models per marker), high baseline GFAP (HR 1.89, 95%CI: 1.47–2.43; model C-index 0.69 (0.63–0.76)) and high baseline pTau181 (HR 1.45 (1.19–1.77); model C-index 0.66 (0.59–0.74)) were associated with incident dementia, whereas NfL and Aβ42/40 were not (Fig. 1, Table 2). Addition of plasma GFAP, pTau181, or all plasma biomarkers improved the model discrimination compared to reference demographics model, with C-index increases of 0.077 (0.027–0.137) for GFAP alone, 0.047 (0.009–0.110) for pTau181 alone, and 0.095 (0.041–0.156) for all biomarkers (Table 2). In the full model including all plasma biomarkers, GFAP and MMSE score were the only variables which remained significantly associated with any-cause dementia risk (Table 2). Forcing age, sex and MMSE in the model, backward selection based on the AIC resulted in a parsimonious model which included age, sex, MMSE and plasma GFAP. For this parsimonious model, the time-dependent Brier accuracy score, ranged from 0.031 (0.030—0.032) at one year, to 0.221 (0.220—0.222) at five years, indicating the ability of the model to make accurate individualised predictions over a timeframe of up to 5 years (Table 2 and eFigure 1).

**Table 2:** Associations of model variables with risk of clinical progression to any-cause dementia from MCI and evaluation of model performances

	Hazard Ratio	Linear Predictor	*P *Value	C-index (95% CI)	C-index difference (95% CI)	1-year Brier score (95% CI)	3-year Brier score (95% CI)	5-year Brier score (95% CI)
Model 1 – Baseline demographics only								
Age	1.00 (0.97–1.03)	0.00	0.983	0.62 (0.56—0.67)	Ref	0.031 (0.031—0.032)	0.203 (0.203—0.204)	0.231 (0.231—0.232)
Sex	1.64 (1.09–2.46)	0.49	0.017					
MMSE Score	0.90 (0.83–0.99)	−0.11	0.024					
Model 2—Baseline demographics + NfL								
Age	0.99 (0.96–1.02)	−0.01	0.565	0.63 (0.57–0.69)	0.012 (−0.005–0.052)	0.031 (0.031—0.032)	0.203 (0.202—0.203)	0.232 (0.231—0.233)
Sex	1.72 (1.14–2.59)	0.54	0.010					
MMSE Score	0.90 (0.83–0.99)	−0.11	0.026					
NfL	1.16 (0.92–1.45)	0.14	0.205					
Model 3—Baseline demographics + Aβ42/40								
Age	1.00 (0.97–1.03)	0.00	0.991	0.63 (0.57–0.68)	0.008 (−0.007–0.039)	0.031 (0.031—0.032)	0.202 (0.202—0.203)	0.235 (0.234—0.236)
Sex	1.54 (1.01–2.34)	0.43	0.044					
MMSE Score	0.89 (0.82–0.98)	−0.11	0.016					
Aβ42/40	0.87 (0.68–1.10)	−0.14	0.238					
Model 4—Baseline demographics + GFAP (parsimonious model)								
Age	0.97 (0.93–1.00)	−0.03	0.029	0.69 (0.63–0.76)	0.077 (0.027–0.137)	0.031 (0.030—0.032)	0.200 (0.199—0.201)	0.221 (0.220—0.222)
Sex	1.59 (1.06–2.40)	0.46	0.025					
MMSE Score	0.89 (0.81–0.97)	−0.12	0.011					
GFAP	1.89 (1.47–2.43)	0.64	< 0.001					
Model 5—Baseline demographics + pTau181								
Age	0.99 (0.96–1.02)	−0.01	0.496	0.66 (0.59–0.74)	0.047 (0.009–0.110)	0.031 (0.031—0.032)	0.201 (0.200—0.202)	0.232 (0.231—0.233)
Sex	1.72 (1.14–2.58)	0.54	0.009					
MMSE Score	0.90 (0.82–0.98)	−0.11	0.019					
pTau181	1.45 (1.19–1.77)	0.37	< 0.001					
Model 6 – Baseline demographics + the panel of all four plasma biomarkers								
Age	0.97 (0.94–1.00)	−0.03	0.074	0.71 (0.65–0.77)	0.095 (0.041–0.156)	0.031 (0.030—0.032)	0.201 (0.200—0.202)	0.226 (0.225—0.228)
Sex	1.47 (0.96–2.26)	0.39	0.079					
MMSE Score	0.88 (0.80–0.96)	−0.13	0.006					
NfL	0.86 (0.65–1.13)	−0.15	0.285					
Aβ42/40	0.90 (0.71–1.14)	−0.11	0.364					
GFAP	1.83 (1.35–2.47)	0.60	< 0.001					
pTau181	1.20 (0.95–1.52)	0.18	0.131					

The table displays hazard ratio (95% CI) for all variables in each model. Biomarker values were natural log-transformed and standardized as Z scores for comparability of effect sizes. The point at which clinical progression occurred was defined as the first visit at which dementia was diagnosed. Harrell’s C-index is used to assess prognostic discrimination and ranges from 0 to 1, with a score of 0.5 indicating risk score predictions are no better than a random prediction, and a score of 1 indicating perfect model prediction. The brier score is used to assess the accuracy of probabilistic predictions at a given time, and ranges from 0 to 1, with a score closer to 0 indicating greater accuracy. Model performance metrics and their 95% CIs were derived using bootstrapping. The parsimonious model was assigned using backward selection based on the AIC, starting with the full model which included age, sex, MMSE score and all four biomarkers. The baseline survival probabilities at 1, 3, and 5 years were 0.98, 0.80, and 0.50, respectively, based on the parsimonious model. Aβ42/40 = Amyloid β42/40. GFAP = glial fibrillary acidic protein. MMSE = mini-mental state examination. NfL = neurofilament light. pTau181 = phosphorylated-tau-181

When examining the association between the plasma biomarkers and risk of progression from MCI to dementia due to AD specifically, demographic and plasma biomarker variables showed similar associations (direction and magnitude) with AD dementia risk compared to any-cause dementia risk, and model discrimination and model calibration were also comparable (eTable 2 and eFigures 2–3). Unlike the parsimonious model with any-cause dementia as outcome, the AD-specific biomarker pTau181 was selected in addition to GFAP for the AD dementia parsimonious model, which had superior discrimination compared to the demographic only model (eTable 2).

We repeated our analysis in a subset of the MCI cohort for which pTau217 measurements were available. This subset was comprised of 197 individuals with a baseline diagnosis of MCI, of which 77 (39%) converted to dementia during follow-up, the majority (92%) of which converted specifically to AD dementia (eTable 3). To maximally leverage available data, subsequent analysis used any-cause dementia as outcome only. High baseline pTau217 was associated with incident dementia when added to a demographics-only prognostic model including age, sex and MMSE score (HR 2.07 (1.57–2.71, eTable 4). Starting from a full model including age, sex, MMSE score, and all plasma biomarkers, backward selection based on the AIC resulted in a parsimonious model which included age, sex, MMSE and only plasma pTau217 (C-index 0.75 (0.69–0.79)). The addition of pTau217 to the demographic-only model significantly improved the model discrimination (C-index difference 0.121 (0.066–0.201), eTable 4). This parsimonious model including pTau217 showed the best predictive accuracy until around 3.5 years, however over a longer time period the model including baseline demographics with GFAP showed superior accuracy (lower 5-year Brier score, eTable 4 and eFigure 4).

For the SCD sample, in the models which included demographics and one plasma biomarker, high baseline GFAP, pTau181 and Aβ42/40, but not NfL, were associated with increased risk of dementia (eTable 5). We could not robustly estimate model discrimination and accuracy performances for the SCD models due to too few individuals with SCD progressing to dementia during follow-up, hence obtaining a parsimonious model was not feasible (eTables 5–6).

### External validation

We performed external validation of our parsimonious models for MCI developed in ADC, on a sample comprising individuals from the MEMENTO study and the AIBL study. Cohort differences included lower MMSE scores at baseline in ADC and AIBL compared to MEMENTO, younger age in ADC compared to AIBL and MEMENTO, more males in ADC compared to AIBL and MEMENTO, higher progression rates in ADC and AIBL compared to MEMENTO and shorter follow-up time in ADC compared to MEMENTO and AIBL (Table 3).

**Table 3:** Baseline demographics and clinical characteristics of the validation cohorts

	ADC *n* = 253	Memento *n* = 782	AIBL *n* = 265
No. with clinical progression to any-cause dementia (%)ᶧ	99 (39%)	130 (17%)	112 (42%)
No. with clinical progression to AD dementia (%)ᶧ	91 (36%)	101 (13%)	104 (39%)
Age, yearsᶧ	66.0 (60.7–71.2)	72.9 (66.8–78.5)	75.9 (71.3–81.0)
Sex, no. males (%)ᶧ	165 (65%)	347 (44%)	141 (53%)
MMSEᶧ	27 (25–28)	28 (26–29)	27 (25–28)
Follow-up duration, yearsᶧ	2.4 (1.2–3.6)	3.0 (2.5–3.1)	3.0 (1.6–4.7)

Data are n (%), or median (IQR) for the total groups. Differences between cohorts were measured with chi-square and ANOVA where applicable. MMSE = mini-mental state examination

ᶧindicates *p* < 0.001

The models showed very good discriminative performance in MEMENTO but poor performance in AIBL (any-cause dementia model including GFAP, Harrell’s C = 0.77 in MEMENTO, 0.56 in AIBL; AD dementia model including pTau181 and GFAP, C = 0.81 in MEMENTO, 0.55 in AIBL; any-cause dementia model including pTau217, C = 0.54 in AIBL, not performed in MEMENTO, eTables 7–9).

Similarly, the parsimonious models showed good calibration in MEMENTO but calibrated less well in AIBL (eTables 7–9): observed and expected survival curves overlapped in MEMENTO while expected risks tended to be under- or overestimated in AIBL (eFigures 5–7).

### Personalised probabilities

To increase the clinical applicability of the prognostic models, we used the parsimonious models to determine personalized risk probabilities for progression to any-cause dementia (age, sex, MMSE and plasma GFAP or when available age, sex, MMSE and plasma pTau217) and AD-dementia (age, sex, MMSE, plasma GFAP and plasma pTau181). These were calculated at 1, 3, and 5 years at the level of the individual.

**Fig. 2:**
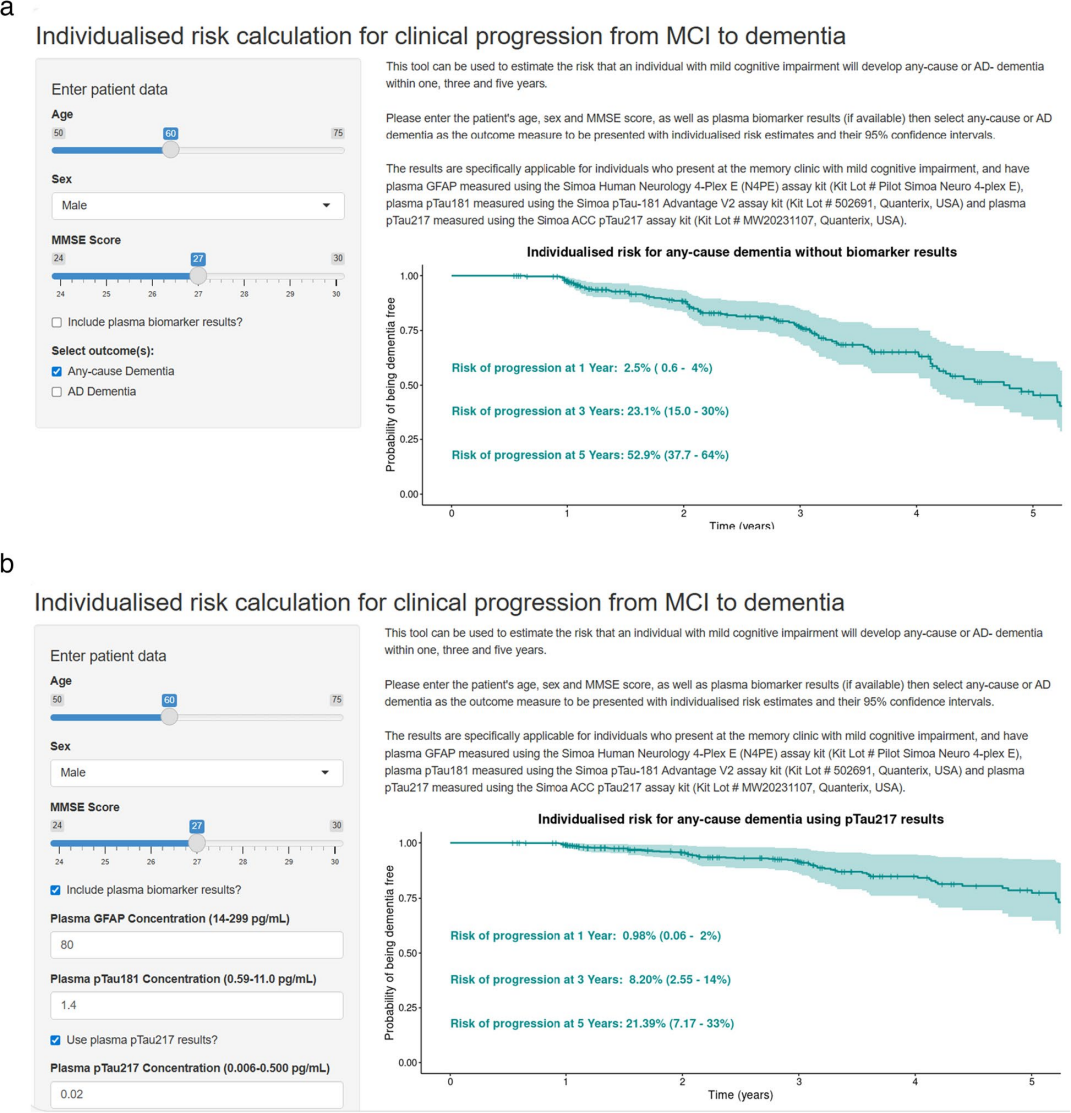
Screenshot of the interactive interface to estimate the risk that an individual with mild cognitive impairment will develop any-cause or AD-dementia within one, three and five years. **a** The demographics-only prognostic model including age, sex and MMSE score is used to calculate the risk probabilities in the absence of biomarker results. **b** The parsimonious models utilising plasma GFAP or either plasma pTau217 alone (for any-cause dementia as outcome), or both GFAP and pTau181 (for AD dementia as outcome) are used to calculate the risk probabilities upon entering the biomarker results of an individual. GFAP = glial fibrillary acidic protein. MMSE = mini-mental state examination. pTau181 = phosphorylated-tau-181. pTau217 = phosphorylated-tau-217. The interface is accessible through the link: https://plasmabiomarkers.shinyapps.io/BiomarkerPredict/

Individualised progression risks can be calculated for any value of plasma biomarker and demographic variables, therefore we developed a publicly accessible interactive app available at https://plasmabiomarkers.shinyapps.io/BiomarkerPredict/ in order to facilitate the use of the prognostic models. After entering the age, sex and MMSE score of a patient, and selecting any-cause or AD- dementia as the outcome, individualised risk of progression to dementia within one, three and five years is displayed (Fig. 2a). If plasma biomarker results are entered, the risk estimates generated from the parsimonious models are shown, with the choice to use GFAP, or alternatively pTau217 results to determine the risk of progression to any-cause dementia, and GFAP and pTau181 to determine the risk of progression to AD dementia (Fig. 2b). To illustrate the added value of plasma biomarkers compared to demographics-only for progression risk estimation, see one example in Fig. 2.

**Fig. 3:**
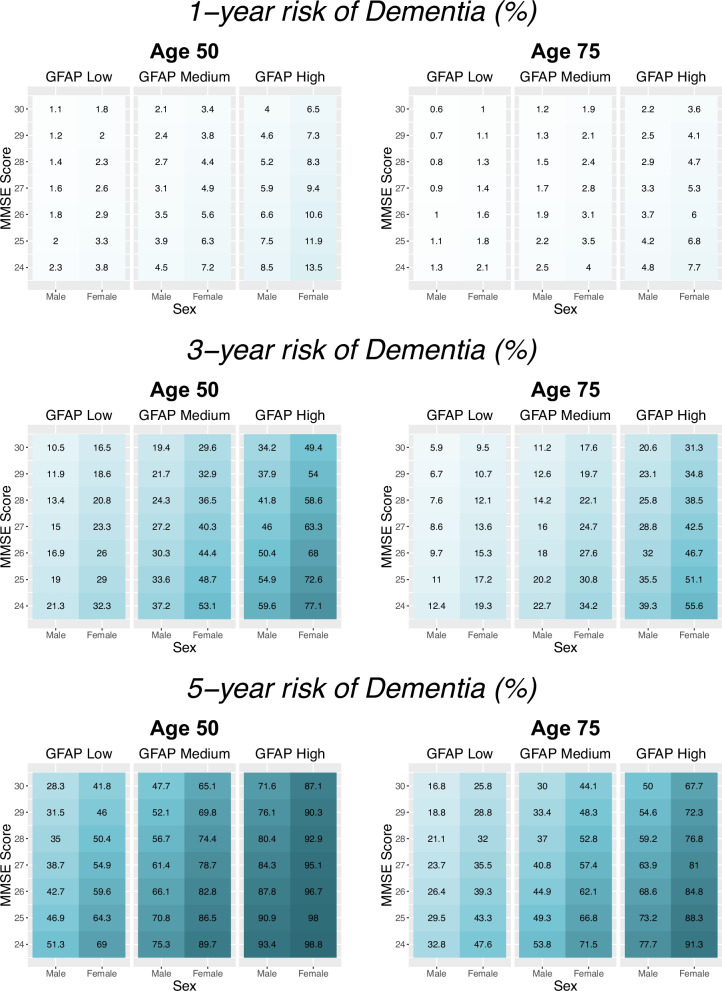
Risk charts to visualize risk of progression in the MCI population to any-cause dementia within 1, 3 and 5 years. The prognostic model used to calculate the risk probabilities included age, sex, MMSE score and plasma GFAP measured at baseline. Plasma GFAP values were natural log-transformed, standardized as Z scores and split into tertiles. As the prognostic model can provide risk estimates for any variable values, these graphs provide an example, with estimates for males and females, age 50 and age 75, with an MMSE score ranging from 24 to 30. GFAP = glial fibrillary acidic protein. MMSE = mini-mental state examination

Although the interactive interface can calculate progression risk estimates for any value of the variables, for visualisation purposes we additionally constructed heatmaps with risk estimates for biomarker tertiles, separated for sex, age and MMSE score (Fig. 3). The risk charts clearly visualize that an individual with a plasma GFAP concentration in the highest tertile has a much larger 1-, 3- and 5- year risk of progression to any-cause dementia than if the same individual would have had a plasma GFAP concentration in the lowest tertile. The risk estimate differences between low and high biomarker results become more pronounced at longer time points (Fig. 3).

## Discussion

In this study we demonstrated that individualized risk prediction of dementia for MCI patients is possible using plasma biomarkers, and that plasma biomarkers have added value on top of demographic information. The individualized risk models we developed validated well in patients with MCI recruited through independent memory clinics (MEMENTO), but may require further calibration for patients with MCI recruited from the general population (AIBL). We found that utilizing plasma pTau217 results in a best performing prognostic model, but in the absence of this biomarker, GFAP and pTau181 could be used to estimate individualized risk to develop dementia in MCI patients. Assessing risk of progression at the level of the individual in early clinical dementia stages using simple baseline demographic information and plasma biomarker concentrations may enable more confident discussion of clinical trajectories with patients. Additionally, it provides patients with answers to questions concerning future progression, which individuals with MCI express a desire to have answered [22–24]. Accurate prediction of dementia risk can also be valuable for clinical trials which target early symptomatic AD, to increase the likelihood of efficacy by treating those individuals at higher risk of progression to dementia [43].

The individualized prognostic value of more extensive testing has been examined previously, using CSF biomarker and imaging results, with models developed in a population of individuals with MCI from the same memory clinic [3, 4, 44]. The C-indices of models predicting any-cause dementia including CSF biomarkers, hippocampal volume or a combination of both measures ranged from 0.69 to 0.72. Other studies have investigated the use of machine learning applied to neuroimaging data for prediction of AD dementia from MCI, which showed a mean accuracy of 75% [45]. Our models demonstrated comparable accuracy, with C-indices which can be considered moderate to good. This further supports the use of our blood biomarker models for prognosis, however, given that the C-indices are not maximal, the models should be interpreted with caution. The models may require further development, such as by addition of other blood-based biomarkers, or by re-training them on our cohort after extended follow-up.

When we evaluated the blood biomarkers individually in our prognostic models, we found specifically that high plasma GFAP, pTau181 and pTau217 were valuable for risk estimation of clinical progression to dementia. Our finding that these biomarkers have prognostic value for progression to dementia is in agreement with former studies [14, 15, 18, 19, 46–48]. We found that plasma NfL had limited utility for dementia prognosis, in contrast to prior studies [13, 16, 49], which may reflect the stronger predictive value of demographics and baseline cognitive performance in our memory-clinic population. The model with plasma pTau217 had the highest discrimination, which is consistent with literature demonstrating the superior prognostic performance of this biomarker [18, 19]. In the subset of MCI individuals with pTau217 measurements, the results are likely to be driven by the high proportion of converters to AD dementia (92% of the total group) during follow-up. The specificity of pTau for AD dementia prognosis is expected in view of its relation to AD neuropathology [50]. Interestingly, in the MCI subset, the model with plasma GFAP had better long-term accuracy when directly compared to pTau217 and all other biomarkers (lowest 5-year Brier score), highlighting its potential value for longer term prediction of dementia. Our findings highlight that GFAP or pTau217 alone have utility for prediction of dementia, and combining pTau and GFAP adds specificity for AD dementia prognosis.

We observed that younger individuals demonstrated a higher risk of progression than older individuals. This may reflect that in midlife individuals, abnormal biomarker profiles are less prevalent and therefore more strongly associated with aggressive disease phenotypes. Whereas in later life, these abnormalities are more common and their predictive value is influenced by competing comorbidities and age-related processes. Given this, our focus was on a younger memory clinic population, in whom biomarker-driven models may be particularly informative.

When we externally validated our models, they showed better discrimination and calibration in the MEMENTO cohort compared to the AIBL cohort. Both cohorts were older on average than the ADC cohort used for model development, however, MEMENTO participants are from memory clinics, while AIBL participants are recruited from the general population. This indicates that the recruitment setting, reflecting the clinical context and underlying risk enrichment, may be the most important factor underlying the generalisability of our models. The less optimal external validation using the AIBL cohort suggests our models will need updating before they are translatable to an aging general population, where disease trajectories are shaped by a broader range of comorbid and age-related factors.

When we assessed the prognostic value of the biomarkers in individuals with SCD, we found that Aβ42/40, GFAP and pTau181 were significantly associated with the risk of clinical progression to dementia. Whereas, for progression to AD dementia specifically, higher baseline NfL, GFAP and pTau181 were significantly associated with an increased risk of clinical progression. The predictive performance of pTau217 for progression in SCD was not assessed. Other studies which have examined the prognostic value of plasma biomarkers in cognitively unimpaired individuals have demonstrated results which are in line with our findings [49, 51]. Despite the statistical significance of biomarker predictors, this did not translate to robust personalised predictions. A study which included a very large number of individuals with SCD demonstrated similar findings using CSF biomarkers, namely that the predictive models converged, but did not validate externally [5]. Our results demonstrating the limited prognostic use of blood biomarkers for individuals with SCD are in line with current recommendations to not perform plasma biomarker testing in clinical practice for this population [10, 52].

We developed an online interface to facilitate use of our models for MCI patients (https://plasmabiomarkers.shinyapps.io/BiomarkerPredict/). Our freely accessible tool estimates the risk that an individual with MCI will develop dementia for up to five years’ time. Our tool differs from a previously published prediction app [46] as we provide progression probabilities for both AD and any-cause dementia, and continuous biomarker values can be entered to obtain personalised risk probabilities as opposed to a binary status. The app provides the option to utilise either pTau217 or GFAP alone, or GFAP in combination with pTau181, to determine the risk of progression to dementia and provides 95% confidence intervals for risk estimates.

## Limitations

Our cohort included all individuals with MCI who entered our clinic with at least one clinical follow-up visit available, rather than those selected for progression to a particular type of dementia, therefore reducing selection bias. However, the follow-up time for these individuals was relatively short (median 2.4 years), therefore the number of individuals observed at later time points was reduced. While the models maintained sufficient performance at five years, the decline in accuracy with time elapsing (higher Brier scores) reflects this smaller sample size. Having a longer follow-up time would allow for more accurate predictions over a longer time period than five years. As pTau217 was only available in a subset of participants, this limited our ability to perform analyses specifically predicting AD dementia. Additionally, we were limited by the low number of individuals with SCD who progressed to dementia during the study follow-up, which is expected given that approximately 11% of individuals with SCD develop dementia over a four year period [53]. The discriminatory power of our models was moderate to good, however, we performed extensive internal and external validation using cohorts from different settings to better understand the robustness and generalisability of our developed models. Lastly, while our models demonstrate prognostic potential, they are not yet intended for routine clinical use. Blood biomarker-based risk prediction is at the research-use stage. To support its integration into clinical guidelines and evidence-based medicine, unselected, prospective studies with clinical follow-up are needed to generate real-world evidence. The online interface we developed is also only applicable for plasma biomarker values measured using the specific kit lot numbers and the Simoa platform used in our development cohort. Work to adapt input values to those obtained with other analytical platforms is part of ongoing efforts, such as round robin studies and the development of certified reference materials and methods. Nonetheless, studies such as ours provide important data that, with further validation and standardization, may contribute to the development of evidence-based guidelines and ultimately support the clinical utility of plasma biomarker-informed risk prediction.

## Conclusions

In conclusion, we developed personalised risk models based on plasma biomarkers to identify individuals with MCI who are at high risk of progression to dementia. Plasma GFAP, pTau181 and/or pTau217 can be utilised to provide complementary information to simple demographic data for generating individualized risk estimates. With more real-world validation, our individualised prognostic models may aid a clinician in providing individual progression risk estimates using the developed interactive app. We envision this approach can improve patient management and pave the way to a new era of accessible precision medicine for individuals in the early symptomatic stages of dementia.

## Supplementary Information

Supplementary Material 1.

## Data Availability

The Amsterdam Dementia Cohort dataset supporting the conclusions of this article is available from the corresponding author upon reasonable research request.

## Abbreviations

Aβ42/40: Amyloid beta1-42/1–40
AD: Alzheimer’s disease
CSF: Cerebrospinal fluid
GFAP: Glial fibrillary acidic protein
HR: Hazard ratio
MCI: Mild cognitive impairment
MMSE: Mini mental state examination
NfL: Neurofilament light
PET: Positron emission tomography
pTau: Phosphorylated-tau
SCD: Subjective cognitive decline
