# Supplementary material for: Individualized prediction of clinical progression to dementia using plasma biomarkers in non-demented elderly
Source: Alzheimers Res Ther. 2025 Dec 3;18:4. doi: 10.1186/s13195-025-01925-1 (PMC12781432; doi:10.1186/s13195-025-01925-1)
Supplement: Supplementary file 1 — Supplementary Material 1. [file 13195_2025_1925_MOESM1_ESM.docx]

**SUPPLEMENTARY FILE**

**Individualized prediction of clinical progression to dementia using plasma biomarkers in non-demented elderly.**

**Contents**

eMethods: TRIPOD checklist for prediction model development and validation.

eTable 1: Baseline demographics and clinical characteristics of the SCD population, stratified for conversion to any-cause dementia at follow-up.

eTable 2: Associations of model variables with risk of clinical progression to Alzheimer’s disease dementia from MCI and evaluation of model performances.

eTable 3: Baseline demographics and clinical characteristics of the MCI population with pTau217 measurements, stratified for conversion to any-cause dementia at follow-up.

eTable 4: Associations of model variables with risk of clinical progression to any-cause dementia in the MCI population with pTau217 measurements and evaluation of model performances.

eTable 5: Associations of model variables with risk of clinical progression to any-cause dementia in the SCD subset.

eTable 6: Associations of model variables with risk of clinical progression to Alzheimer’s disease dementia in the SCD subset.

eTable 7: Harrell’s C-index and hazard ratios evaluated in the ADC and validation datasets, for the any-cause dementia prognostic model using GFAP.

eTable 8: Harrell’s C-index and hazard ratios evaluated in the ADC and AIBL validation dataset, for the any cause dementia prognostic model using pTau217.

eTable 9: Harrell’s C-index and hazard ratios evaluated in the ADC and validation datasets, for the AD dementia prognostic model using GFAP and pTau181.

eFigure 1: Brier scores of prognostic models over time for any-cause dementia as outcome.

eFigure 2: Kaplan-Meier curves of clinical progression to AD dementia for individuals with low, medium, or high baseline plasma biomarker levels in the MCI subset.

eFigure 3: Brier scores of prognostic models over time for AD dementia as outcome.

eFigure 4: Brier scores of prognostic models over time for any cause dementia as outcome, including pTau217 data.

eFigure 5: Calibration of GFAP model performance for any-cause dementia as a clinical endpoint in ADC (left) MEMENTO (middle) and AIBL (right) cohorts.

eFigure 6: Calibration of pTau217 model performance for any cause dementia as a clinical endpoint in ADC (left) and AIBL (right) cohorts.

eFigure 7: Calibration of GFAP and pTau181 model performance for AD dementia as a clinical endpoint in ADC (left) MEMENTO (middle) and AIBL (right) cohorts.

**eMethods: TRIPOD checklist for prediction model development and validation.**

| **Section/Topic** | **Item** |  | **Checklist Item** | **Included?** |
| --- | --- | --- | --- | --- |
| **Title and abstract** | | | | |
| Title | 1 | D;V | Identify the study as developing and/or validating a multivariable prediction model, the target population, and the outcome to be predicted. | Yes, title |
| Abstract | 2 | D;V | Provide a summary of objectives, study design, setting, participants, sample size, predictors, outcome, statistical analysis, results, and conclusions. | Yes, abstract |
| **Introduction** | | | | |
| Background and objectives | 3a | D;V | Explain the medical context (including whether diagnostic or prognostic) and rationale for developing or validating the multivariable prediction model, including references to existing models. | Yes, background |
|  | 3b | D;V | Specify the objectives, including whether the study describes the development or validation of the model or both. | Yes, background |
| **Methods** | | | | |
| Source of data | 4a | D;V | Describe the study design or source of data (e.g., randomized trial, cohort, or registry data), separately for the development and validation data sets, if applicable. | Yes, methods 2.1 and 2.4 |
|  | 4b | D;V | Specify the key study dates, including start of accrual; end of accrual; and, if applicable, end of follow-up. | Yes, methods 2.1 and 2.4 |
| Participants | 5a | D;V | Specify key elements of the study setting (e.g., primary care, secondary care, general population) including number and location of centres. | Yes, methods 2.1 and 2.4 |
|  | 5b | D;V | Describe eligibility criteria for participants. | Yes, methods 2.1 and 2.4 |
|  | 5c | D;V | Give details of treatments received, if relevant. | NA |
| Outcome | 6a | D;V | Clearly define the outcome that is predicted by the prediction model, including how and when assessed. | Yes, methods 2.2 and 2.4 |
|  | 6b | D;V | Report any actions to blind assessment of the outcome to be predicted. | Yes, methods 2.2 and 2.4 |
| Predictors | 7a | D;V | Clearly define all predictors used in developing or validating the multivariable prediction model, including how and when they were measured. | Yes, methods 2.3 – 2.5 |
|  | 7b | D;V | Report any actions to blind assessment of predictors for the outcome and other predictors. | Yes, methods 2.3 – 2.4 |
| Sample size | 8 | D;V | Explain how the study size was arrived at. | Yes, methods 2.1 and 2.4 |
| Missing data | 9 | D;V | Describe how missing data were handled (e.g., complete-case analysis, single imputation, multiple imputation) with details of any imputation method. | Yes, methods 2.5 |
| Statistical analysis methods | 10a | D | Describe how predictors were handled in the analyses. | Yes, methods 2.5 |
|  | 10b | D | Specify type of model, all model-building procedures (including any predictor selection), and method for internal validation. | Yes, methods 2.5 |
|  | 10c | V | For validation, describe how the predictions were calculated. | Yes, methods 2.4 |
|  | 10d | D;V | Specify all measures used to assess model performance and, if relevant, to compare multiple models. | Yes, methods 2.5 |
|  | 10e | V | Describe any model updating (e.g., recalibration) arising from the validation, if done. | NA |
| Risk groups | 11 | D;V | Provide details on how risk groups were created, if done. | Yes, methods 2.4 |
| Development vs. validation | 12 | V | For validation, identify any differences from the development data in setting, eligibility criteria, outcome, and predictors. | Yes, methods 2.4 |
| **Results** | | | | |
| Participants | 13a | D;V | Describe the flow of participants through the study, including the number of participants with and without the outcome and, if applicable, a summary of the follow-up time. A diagram may be helpful. | Yes, table 1, table 3 and supplement |
|  | 13b | D;V | Describe the characteristics of the participants (basic demographics, clinical features, available predictors), including the number of participants with missing data for predictors and outcome. | Yes, table 1, table 3 and supplement |
|  | 13c | V | For validation, show a comparison with the development data of the distribution of important variables (demographics, predictors and outcome). | Yes, table 3 and results 3.2 |
| Model development | 14a | D | Specify the number of participants and outcome events in each analysis. | Yes, results 3.1 and supplement |
|  | 14b | D | If done, report the unadjusted association between each candidate predictor and outcome. | NA |
| Model specification | 15a | D | Present the full prediction model to allow predictions for individuals (i.e., all regression coefficients, and model intercept or baseline survival at a given time point). | Yes, table 2 and supplement |
|  | 15b | D | Explain how to the use the prediction model. | Yes, results 3.3 |
| Model performance | 16 | D;V | Report performance measures (with CIs) for the prediction model. | Yes, table 2 and supplement |
| Model-updating | 17 | V | If done, report the results from any model updating (i.e., model specification, model performance). | NA |
| **Discussion** | | | | |
| Limitations | 18 | D;V | Discuss any limitations of the study (such as nonrepresentative sample, few events per predictor, missing data). | Yes, limitations |
| Interpretation | 19a | V | For validation, discuss the results with reference to performance in the development data, and any other validation data. | Yes, discussion |
|  | 19b | D;V | Give an overall interpretation of the results, considering objectives, limitations, results from similar studies, and other relevant evidence. | Yes, discussion |
| Implications | 20 | D;V | Discuss the potential clinical use of the model and implications for future research. | Yes, discussion |
| **Other information** | | | | |
| Supplementary information | 21 | D;V | Provide information about the availability of supplementary resources, such as study protocol, Web calculator, and data sets. | Yes, declarations |
| Funding | 22 | D;V | Give the source of funding and the role of the funders for the present study. | Yes, declarations |

D = items relevant to prediction model development, V = items relevant to prediction model validation.

**eTable 1: Baseline demographics and clinical characteristics of the SCD population, stratified for conversion to any-cause dementia at follow-up.**

|  | **Total group** | **Stratified for diagnosis at follow-up** | |
| --- | --- | --- | --- |
|  |  | **Stable** | **Progression to dementia** |
| **Number of participants** | 314 (100%) | 294 (94%) | 20 (6%) |
| **Demographic and clinical characteristics** | | | |
| **Age, years** | 61 (9) | 61 (9) | 67 (7) |
| **Sex** | | | |
| **Male** | 184 (59%) | 172 (59%) | 12 (60%) |
| **Education (range 1-7)** | 6 (5-6) | 6 (5-6) | 6 (5-7) |
| **Follow-up duration, years** | 4.1 (2.6 – 5.5) | 4.1 (2.5 – 5.4) | 4.0 (3.0 – 6.1) |
| **Number of visits** | 4 (3 - 5) | 4 (3 – 5) | 4 (3 – 6) |
| **Time to progression, years** | --- | --- | 4.0 (2.7 – 6.1) |
| **Cognitive test performance** | | | |
| **MMSE** | 29 (28 – 30) | 29 (28 – 30) | 28 (27 – 29) |
| **Plasma biomarkers** | | | |
| **NfL (pg/mL)** | 11.2 (8.6 – 15.6) | 10.9 (8.50 – 14.8) | 16.0 (12.5 – 22.0) |
| **Aβ42/40** | 0.058 (0.052 – 0.063) | 0.058 (0.052 – 0.063) | 0.051 (0.048 – 0.054) |
| **GFAP (pg/mL)** | 67.6 (48.5 – 96.6) | 66.2 (47.8 – 92.2) | 112.8 (78.1 – 166.3) |
| **pTau181 (pg/mL)** | 1.36 (1.07 – 1.96) | 1.33 (1.04 – 1.82) | 2.05 (1.61 – 2.55) |

Data are n (%), mean (SD), or median (IQR) for the total group and stratified for individuals who received a diagnosis of dementia during follow-up or those who remained non-demented at their last visit. The group who progressed to dementia during follow-up was comprised of 12 Alzheimer’s Disease, 3 Dementia with Lewy Bodies, 3 vascular dementia and 2 Progressive supranuclear palsy cases. Aβ42/40 = Amyloid β42/40. GFAP = glial fibrillary acidic protein. MMSE = mini-mental state examination. NfL = neurofilament light. pTau181= phosphorylated-tau-181.

**eTable 2: Associations of model variables with risk of clinical progression to Alzheimer’s disease dementia from MCI and evaluation of model performances.**

|  | **Hazard Ratio** | **Linear Predictor** | ***P* Value** | **C-index (95% CI)** | **C-index difference (95% CI)** | **1-year Brier score (95% CI)** | **3-year Brier score (95% CI)** | **5-year Brier score (95% CI)** |
| --- | --- | --- | --- | --- | --- | --- | --- | --- |
| **Model 1 – Baseline demographics only** | | | | | | | | |
| Age | 1.00 (0.97 – 1.03) | 0.00 | 0.781 | 0.61 (0.55 - 0.66) | Ref | 0.032 (0.031 - 0.032) | 0.204 (0.203 - 0.204) | 0.242 (0.241 - 0.243) |
| Sex | 1.73 (1.14 – 2.63) | 0.55 | 0.010 |  |  |  |  |  |
| MMSE Score | 0.90 (0.82 – 0.99) | -0.11 | 0.025 |  |  |  |  |  |
| **Model 2 -**  **Baseline demographics + NfL** | | | | | | | | |
| Age | 0.98 (0.95 – 1.02) | -0.02 | 0.320 | 0.63 (0.56 – 0.68) | 0.012 (-0.004 – 0.054) | 0.032 (0.031 - 0.032) | 0.204 (0.203 - 0.205) | 0.244 (0.243 - 0.245) |
| Sex | 1.85 (1.21 – 2.83) | 0.61 | 0.005 |  |  |  |  |  |
| MMSE Score | 0.90 (0.82 – 0.99) | -0.11 | 0.026 |  |  |  |  |  |
| NfL | 1.22 (0.98 – 1.54) | 0.20 | 0.081 |  |  |  |  |  |
| **Model 3 - Baseline demographics + Aβ42/40** | | | | | | | | |
| Age | 1.00 (0.97 – 1.03) | 0.00 | 0.797 | 0.63 (0.56 – 0.69) | 0.014 (-0.014 – 0.066) | 0.032 (0.031 - 0.032) | 0.202 (0.201 - 0.202) | 0.247 (0.246 - 0.248) |
| Sex | 1.53 (0.99 – 2.37) | 0.42 | 0.058 |  |  |  |  |  |
| MMSE Score | 0.89 (0.81 – 0.97) | -0.12 | 0.012 |  |  |  |  |  |
| Aβ42/40 | 0.76 (0.60 – 0.97) | -0.27 | 0.030 |  |  |  |  |  |
| **Model 4 - Baseline demographics + GFAP** | | | | | | | | |
| Age | 0.96 (0.93 – 1.00) | -0.04 | 0.024 | 0.69 (0.64 – 0.75) | 0.083 (0.032 – 0.141) | 0.031 (0.030 - 0.032) | 0.199 (0.199 - 0.200) | 0.227 (0.226 - 0.228) |
| Sex | 1.63 (1.07 – 2.49) | 0.49 | 0.024 |  |  |  |  |  |
| MMSE Score | 0.89 (0.81 – 0.97) | -0.12 | 0.012 |  |  |  |  |  |
| GFAP | 1.99 (1.51 – 2.63) | 0.69 | <0.001 |  |  |  |  |  |
| **Model 5 - Baseline demographics + pTau181** | | | | | | | | |
| Age | 0.99 (0.96 – 1.02) | -0.02 | 0.339 | 0.67 (0.61 – 0.74) | 0.056 (0.014 – 0.116) | 0.031 (0.031 - 0.032) | 0.197 (0.196 - 0.197) | 0.236 (0.235 - 0.237) |
| Sex | 1.84 (1.20 – 2.80) | 0.61 | 0.005 |  |  |  |  |  |
| MMSE Score | 0.89 (0.81 – 0.98) | -0.11 | 0.018 |  |  |  |  |  |
| pTau181 | 1.55 (1.26 – 1.91) | 0.44 | <0.001 |  |  |  |  |  |
| **Model 6 – Baseline demographics + the panel of all four plasma biomarkers** | | | | | | | | |
| Age | 0.96 (0.93 – 1.00) | -0.03 | 0.036 | 0.72 (0.66 – 0.77) | 0.106 (0.041 – 0.172) | 0.031 (0.030 - 0.032) | 0.197 (0.197 - 0.198) | 0.232 (0.231 - 0.233) |
| Sex | 1.50 (0.96 – 2.35) | 0.41 | 0.077 |  |  |  |  |  |
| MMSE Score | 0.87 (0.79 – 0.95) | -0.14 | 0.004 |  |  |  |  |  |
| NfL | 0.91 (0.68 – 1.21) | -0.13 | 0.509 |  |  |  |  |  |
| Aβ42/40 | 0.80 (0.62 – 1.02) | -0.20 | 0.070 |  |  |  |  |  |
| GFAP | 1.80 (1.32 – 2.47) | 0.60 | <0.001 |  |  |  |  |  |
| pTau181 | 1.29 (1.01 – 1.65) | 0.25 | 0.041 |  |  |  |  |  |
| **Model 7 – Parsimonious model** | | | | | | | | |
| Age | 0.96 (0.93 – 0.99) | -0.04 | 0.017 | 0.71 (0.65 – 0.76) | 0.090 (0.034 – 0.158) | 0.031 (0.031 - 0.031) | 0.196 (0.196 - 0.197) | 0.231 (0.230 - 0.232) |
| Sex | 1.70 (1.11 – 2.60) | 0.53 | 0.015 |  |  |  |  |  |
| MMSE Score | 0.88 (0.80 – 0.97) | -0.13 | 0.009 |  |  |  |  |  |
| GFAP | 1.79 (1.34 – 2.40) | 0.58 | <0.001 |  |  |  |  |  |
| pTau181 | 1.29 (1.01 – 1.64) | 0.25 | 0.041 |  |  |  |  |  |

The table displays hazard ratio (95% CI) for all variables in each model. Biomarker values were natural log-transformed and standardized as Z scores for comparability of effect sizes. The point at which clinical progression occurred was defined as the first visit at which Alzheimer’s disease dementia was diagnosed. Harrell’s C-index is used to assess prognostic discrimination and ranges from 0 to 1, with a score of 0.5 indicating risk score predictions are no better than a random prediction, and a score of 1 indicating perfect model prediction. The brier score is used to assess the accuracy of probabilistic predictions at a given time, and ranges from 0 to 1, with a score closer to 0 indicating greater accuracy. The parsimonious model was assigned using backward selection based on the AIC, starting with the full model which included age, sex, MMSE score and all four biomarkers. The baseline survival probabilities at 1, 3, and 5 years were 0.98, 0.82, and 0.57, respectively, based on the parsimonious model. Aβ42/40 = Amyloid β42/40. GFAP = glial fibrillary acidic protein. MMSE = mini-mental state examination. NfL = neurofilament light. pTau181= phosphorylated-tau-181.

**eTable 3: Baseline demographics and clinical characteristics of the MCI population with pTau217 measurements, stratified for conversion to any-cause dementia at follow-up.**

|  | **Total group** | **Stratified for diagnosis at follow-up** | |
| --- | --- | --- | --- |
|  |  | **Stable** | **Progression to dementia** |
| **Number of participants** | 197 (100%) | 120 (61%) | 77 (39%) |
| **Demographic and clinical characteristics** | | | |
| **Age, years** | 65 (7) | 65 (8) | 65 (7) |
| **Sex** | | | |
| **Male** | 127 (64%) | 85 (71%) | 42 (55%) |
| **Education (range 1-7)** | 5 (4-6) | 5 (4-6) | 6 (4-6) |
| **Follow-up duration, years** | 2.3 (1.2 – 3.4) | 2.1 (1.1 – 3.1) | 2.8 (1.5 – 3.6) |
| **Number of visits** | 3 (2 - 4) | 3 (2 - 4) | 3 (2 - 4) |
| **Time to progression, years** | --- | --- | 2.4 (1.5 – 3.5) |
| **Cognitive test performance** | | | |
| **MMSE** | 27 (25 – 28) | 27 (26 – 28) | 26 (25 – 28) |
| **Plasma biomarkers** | | | |
| **NfL (pg/mL)** | 14.0 (10.9 – 18.4) | 13.4 (10.6 – 17.3) | 15.8 (11.5 – 20.6) |
| **Aβ42/40** | 0.053 (0.047 – 0.059) | 0.054 (0.048 – 0.061) | 0.051 (0.047 – 0.056) |
| **GFAP (pg/mL)** | 92.3 (66.1 – 129.6) | 79.8 (55.2 – 106.0) | 120.3 (95.2 – 142.7) |
| **pTau181 (pg/mL)** | 1.90 (1.32 – 2.46) | 1.56 (1.14 – 2.10) | 2.38 (1.96 – 2.81) |
| **pTau217 (pg/mL)** | 0.056 (0.032 – 0.093) | 0.039 (0.028 – 0.065) | 0.097 (0.070 – 0.124) |

Data are n (%), mean (SD), or median (IQR) for the total group and stratified for individuals who received a diagnosis of dementia during follow-up or those who remained non-demented at their last visit. The group who progressed to dementia during follow-up was comprised of 71 AD dementia, 1 Dementia with Lewy Bodies, 3 vascular dementia, 1 frontotemporal dementia and 1 mixed-cause dementia cases. Aβ42/40 = Amyloid β42/40. GFAP = glial fibrillary acidic protein. MMSE = mini-mental state examination. NfL = neurofilament light. pTau181= phosphorylated-tau-181. pTau217= phosphorylated-tau-217.

**eTable 4: Associations of model variables with risk of clinical progression to any-cause dementia in the MCI population with pTau217 measurements and evaluation of model performances.**

|  | **Hazard Ratio** | **Linear Predictor** | ***P* Value** | **C-index (95% CI)** | **C-index difference (95% CI)** | **1-year Brier score (95% CI)** | **3-year Brier score (95% CI)** | **5-year Brier score (95% CI)** |
| --- | --- | --- | --- | --- | --- | --- | --- | --- |
| **Model 1 – Baseline demographics only** | | | | | | | | |
| Age | 1.00 (0.97 – 1.04) | 0.00 | 0.822 | 0.63 (0.55 - 0.68) | Ref | 0.040 (0.040 - 0.041) | 0.213 (0.212 - 0.214) | 0.233 (0.231 - 0.235) |
| Sex | 1.55 (0.98 – 2.46) | 0.44 | 0.059 |  |  |  |  |  |
| MMSE Score | 0.91 (0.83 – 1.00) | -0.09 | 0.059 |  |  |  |  |  |
| **Model 2 -**  **Baseline demographics + NfL** | | | | | | | | |
| Age | 0.99 (0.96 – 1.03) | -0.01 | 0.780 | 0.64 (0.57 – 0.70) | 0.013 (-0.006 – 0.059) | 0.040 (0.040 - 0.041) | 0.211 (0.210 - 0.212) | 0.236 (0.234 - 0.237) |
| Sex | 1.60 (1.01 – 2.54) | 0.47 | 0.046 |  |  |  |  |  |
| MMSE Score | 0.91 (0.82 – 1.00) | -0.10 | 0.056 |  |  |  |  |  |
| NfL | 1.14 (0.89 – 1.46) | 0.13 | 0.303 |  |  |  |  |  |
| **Model 3 - Baseline demographics + Aβ42/40** | | | | | | | | |
| Age | 1.00 (0.97 – 1.04) | 0.00 | 0.959 | 0.64 (0.56 – 0.69) | 0.005 (-0.015 – 0.061) | 0.041 (0.040 - 0.041) | 0.212 (0.211 - 0.213) | 0.237 (0.235 - 0.239) |
| Sex | 1.43 (0.89 – 2.29) | 0.36 | 0.137 |  |  |  |  |  |
| MMSE Score | 0.89 (0.81 – 0.99) | -0.11 | 0.027 |  |  |  |  |  |
| Aβ42/40 | 0.81 (0.62 – 1.05) | -0.22 | 0.111 |  |  |  |  |  |
| **Model 4 - Baseline demographics + GFAP** | | | | | | | | |
| Age | 0.97 (0.94 – 1.01) | -0.03 | 0.126 | 0.70 (0.63 – 0.76) | 0.076 (0.024 – 0.143) | 0.040 (0.039 - 0.040) | 0.209 (0.207 - 0.210) | 0.226 (0.224 - 0.228) |
| Sex | 1.43 (0.90 – 2.26) | 0.36 | 0.129 |  |  |  |  |  |
| MMSE Score | 0.90 (0.81 – 0.99) | -0.11 | 0.036 |  |  |  |  |  |
| GFAP | 2.01 (1.49 – 2.70) | 0.70 | <0.001 |  |  |  |  |  |
| **Model 5 - Baseline demographics + pTau181** | | | | | | | | |
| Age | 0.99 (0.96 – 1.03) | -0.01 | 0.703 | 0.69 (0.61 – 0.75) | 0.058 (0.010 – 0.112) | 0.040 (0.040 - 0.041) | 0.207 (0.207 - 0.208) | 0.239 (0.237 - 0.240) |
| Sex | 1.53 (0.97 – 2.43) | 0.43 | 0.069 |  |  |  |  |  |
| MMSE Score | 0.91 (0.83 – 1.01) | -0.09 | 0.077 |  |  |  |  |  |
| pTau181 | 1.60 (1.27 – 2.02) | 0.47 | <0.001 |  |  |  |  |  |
| **Model 6 – Baseline demographics + pTau217 (parsimonious model)** | | | | | | | | |
| Age | 1.00 (0.96 – 1.03) | 0.00 | 0.831 | 0.75 (0.69 – 0.79) | 0.121 (0.066 – 0.201) | 0.040 (0.039 - 0.040) | 0.203 (0.202 - 0.204) | 0.236 (0.235 - 0.238) |
| Sex | 1.32 (0.83 – 2.10) | 0.27 | 0.248 |  |  |  |  |  |
| MMSE Score | 0.87 (0.78-0.96) | -0.14 | 0.007 |  |  |  |  |  |
| pTau217 | 2.07 (1.57 – 2.71) | 0.73 | <0.001 |  |  |  |  |  |

The table displays hazard ratio (95% CI) for all variables in each model. Biomarker values were natural log-transformed and standardized as Z scores for comparability of effect sizes. The point at which clinical progression occurred was defined as the first visit at which dementia was diagnosed. Harrell’s C-index is used to assess prognostic discrimination and ranges from 0 to 1, with a score of 0.5 indicating risk score predictions are no better than a random prediction, and a score of 1 indicating perfect model prediction. The brier score is used to assess the accuracy of probabilistic predictions at a given time, and ranges from 0 to 1, with a score closer to 0 indicating greater accuracy. Model performance metrics and their 95% CIs were derived using bootstrapping. The parsimonious model was assigned using backward selection based on the AIC, starting with the full model which included age, sex, MMSE score and all five plasma biomarkers. The baseline survival probabilities at 1, 3, and 5 years were 0.97, 0.78, and 0.50, respectively, based on the parsimonious model. Aβ42/40 = Amyloid β42/40. GFAP = glial fibrillary acidic protein. MMSE = mini-mental state examination. NfL = neurofilament light. pTau181= phosphorylated-tau-181. pTau217= phosphorylated-tau-217.

**eTable 5: Associations of model variables with risk of clinical progression to any-cause dementia in the SCD subset**.

|  | **Hazard Ratio** | ***P* Value** |
| --- | --- | --- |
| **Model 1 – Baseline demographics only** | | |
| Age | 1.11 (1.04 – 1.19) | <0.001 |
| Sex | 1.17 (0.47 – 2.93) | 0.739 |
| MMSE | 0.74 (0.57 – 0.96) | 0.021 |
| **Model 2 – Baseline demographics + NfL** | | |
| Age | 1.07 (1.00 – 1.16) | 0.062 |
| Sex | 1.17 (0.47 – 2.94) | 0.736 |
| MMSE | 0.74 (0.57 – 0.96) | 0.021 |
| NfL | 1.68 (0.94 – 3.00) | 0.082 |
| **Model 3 – Baseline demographics + Aβ42/40** | | |
| Age | 1.11 (1.04 – 1.18) | 0.003 |
| Sex | 1.44 (0.56 – 3.71) | 0.453 |
| MMSE | 0.70 (0.54 – 0.92) | 0.011 |
| Aβ42/40 | 0.60 (0.42 – 0.87) | 0.007 |
| **Model 4 – Baseline demographics + GFAP** | | |
| Age | 1.06 (0.99 – 1.14) | 0.088 |
| Sex | 1.00 (0.40 – 2.53) | 0.995 |
| MMSE | 0.78 (0.60 – 1.00) | 0.053 |
| GFAP | 2.70 (1.48 – 4.92) | 0.001 |
| **Model 5 – Baseline demographics + pTau181** | | |
| Age | 1.08 (1.01 – 1.15) | 0.035 |
| Sex | 1.06 (0.42 – 2.64) | 0.903 |
| MMSE | 0.70 (0.53 – 0.93) | 0.013 |
| pTau181 | 1.98 (1.19 – 3.31) | 0.009 |

The table displays hazard ratio (95% CI) for all variables in each model. Biomarker values were natural log-transformed and standardized as Z scores. The point at which clinical progression occurred was defined as the first visit at which dementia was diagnosed. Aβ42/40 = Amyloid β42/40. GFAP = glial fibrillary acidic protein. MMSE = mini-mental state examination. NfL = neurofilament light. pTau181= phosphorylated-tau-181.

**eTable 6: Associations of model variables with risk of clinical progression to Alzheimer’s disease dementia in the SCD subset**.

|  | **Hazard Ratio** | ***P* Value** |
| --- | --- | --- |
| **Model 1 – Baseline demographics only** | | |
| Age | 1.10 (1.01 – 1.19) | 0.020 |
| Sex | 1.88 (0.58 – 6.12) | 0.295 |
| MMSE | 0.66 (0.50 – 0.89) | 0.006 |
| **Model 2 – Baseline demographics + NfL** | | |
| Age | 1.03 (0.94 – 1.14) | 0.515 |
| Sex | 2.03 (0.61 – 6.76) | 0.250 |
| MMSE | 0.65 (0.49 – 0.87) | 0.004 |
| NfL | 2.22 (1.05 – 4.69) | 0.036 |
| **Model 3 – Baseline demographics + Aβ42/40** | | |
| Age | 1.09 (1.00 – 1.18) | 0.044 |
| Sex | 2.26 (0.67 – 7.68) | 0.190 |
| MMSE | 0.64 (0.47 – 0.89) | 0.004 |
| Aβ42/40 | 0.60 (0.33 – 1.08) | 0.089 |
| **Model 4 – Baseline demographics + GFAP** | | |
| Age | 1.03 (0.95 – 1.12) | 0.483 |
| Sex | 1.81 (0.53 – 6.13) | 0.343 |
| MMSE | 0.70 (0.53 – 0.93) | 0.014 |
| GFAP | 3.82 (1.69 – 8.61) | 0.001 |
| **Model 5 – Baseline demographics + pTau181** | | |
| Age | 1.04 (0.96 – 1.13) | 0.365 |
| Sex | 1.74 (0.54 – 5.66) | 0.354 |
| MMSE | 0.57 (0.40 – 0.81) | 0.002 |
| pTau181 | 2.93 (1.52 – 5.64) | 0.001 |

The table displays hazard ratio (95% CI) for all variables in each model. Biomarker values were natural log-transformed and standardized as Z scores. The point at which clinical progression occurred was defined as the first visit at which Alzheimer’s disease dementia was diagnosed. Aβ42/40 = Amyloid β42/40. GFAP = glial fibrillary acidic protein. MMSE = mini-mental state examination. NfL = neurofilament light. pTau181= phosphorylated-tau-181.

**eTable 7: Harrell’s C-index and hazard ratios evaluated in the ADC and validation datasets, for the any-cause dementia prognostic model using GFAP.**

|  | **ADC** | | | **Memento** | | | **AIBL** | | |
| --- | --- | --- | --- | --- | --- | --- | --- | --- | --- |
|  | **Estimate** | **CI** | ***p*** | **Estimate** | **CI** | ***p*** | **Estimate** | **CI** | ***p*** |
| Harrell’s c-index | 0.69 |  |  | 0.77 |  |  | 0.56 |  |  |
| HR: group 2 versus 1 | 2.51 | 0.87 – 7.22 | 0.088 | 1.37 | 0.54 – 3.48 | 0.505 | 1.62 | 0.87 – 3.04 | 0.131 |
| HR: group 3 versus 1 | 4.91 | 1.75 – 13.72 | **0.002** | 3.77 | 1.61 – 8.87 | **0.002** | 1.76 | 0.93 – 3.31 | 0.081 |
| HR: group 4 versus 1 | 9.81 | 3.39 – 28.33 | **<0.001** | 14.70 | 6.36 – 33.97 | **<0.001** | 2.64 | 1.32 – 5.27 | **0.006** |

All values are based on the prognostic index of the prognostic model. The risk groups were defined as: good prognosis (>84th percentile, group 1), fairly good prognosis (50–84th percentile, group 2), fairly poor prognosis (16–50th percentile, group 3), and poor prognosis (<16th percentile, group 4). HR = hazard ratio.

**eTable 8: Harrell’s C-index and hazard ratios evaluated in the ADC and AIBL validation dataset, for the any cause dementia prognostic model using pTau217.**

|  | **ADC** | | | **AIBL** | | |
| --- | --- | --- | --- | --- | --- | --- |
|  | **Estimate** | **CI** | ***p*** | **Estimate** | **CI** | ***p*** |
| Harrell’s c-index | 0.75 |  |  | 0.54 |  |  |
| HR: group 2 versus 1 | 0.51 | 0.17 – 1.53 | 0.229 | 0.72 | 0.40 – 1.30 | 0.281 |
| HR: group 3 versus 1 | 2.22 | 0.86 – 5.70 | 0.098 | 0.86 | 0.49 – 1.53 | 0.616 |
| HR: group 4 versus 1 | 4.67 | 1.79 – 12.18 | 0.002 | 1.34 | 0.70 – 2.57 | 0.377 |

All values are based on the prognostic index of the prognostic model. The risk groups were defined as: good prognosis (>84th percentile, group 1), fairly good prognosis (50–84th percentile, group 2), fairly poor prognosis (16–50th percentile, group 3), and poor prognosis (<16th percentile, group 4). HR = hazard ratio.

**eTable 9: Harrell’s C-index and hazard ratios evaluated in the ADC and validation datasets, for the AD dementia prognostic model using GFAP and pTau181.**

|  | **ADC** | | | **Memento** | | | **AIBL** | | |
| --- | --- | --- | --- | --- | --- | --- | --- | --- | --- |
|  | **Estimate** | **CI** | ***p*** | **Estimate** | **CI** | ***p*** | **Estimate** | **CI** | ***p*** |
| Harrell’s c-index | 0.71 |  |  | 0.81 |  |  | 0.55 |  |  |
| HR: group 2 versus 1 | 1.73 | 0.73 – 4.14 | 0.215 | 0.77 | 0.17 – 3.44 | 0.733 | 0.78 | 0.43 – 1.40 | 0.403 |
| HR: group 3 versus 1 | 2.81 | 1.26 – 6.27 | **0.012** | 7.56 | 2.65 – 21.56 | **<0.001** | 1.29 | 0.74 – 2.25 | 0.372 |
| HR: group 4 versus 1 | 6.36 | 2.99 – 13.54 | **<0.001** | 20.69 | 7.54 – 56.78 | **<0.001** | 1.34 | 0.77 – 2.33 | 0.293 |

All values are based on the prognostic index of the prognostic model. The risk groups were defined as: good prognosis (>75th percentile, group 1), fairly good prognosis (50–75th percentile, group 2), fairly poor prognosis (25–50th percentile, group 3), and poor prognosis (<25th percentile, group 4). HR = hazard ratio.

**eFigure 1: Brier scores of prognostic models over time for any-cause dementia as outcome.**


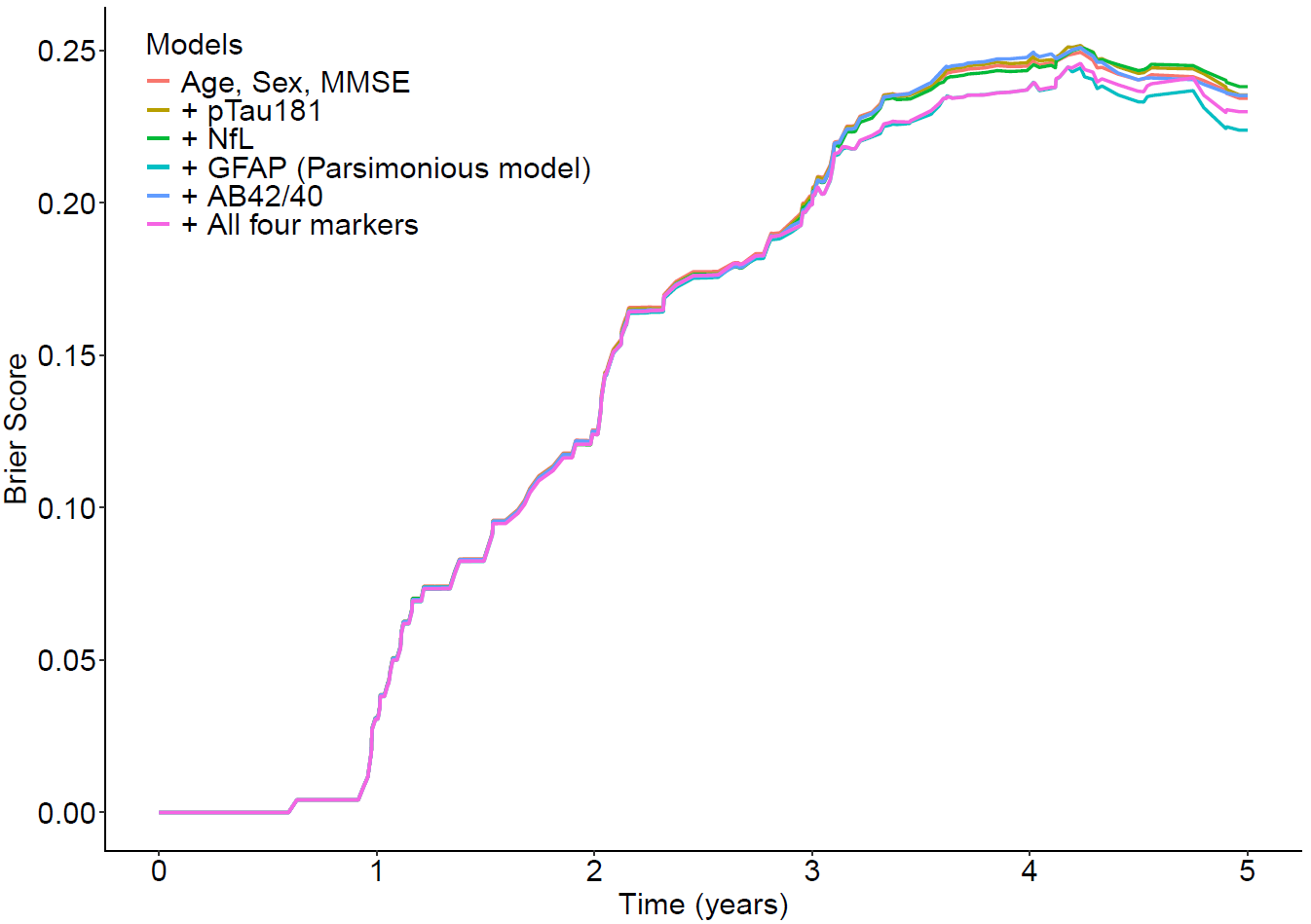


Evaluation of different cox regression prognostic models for any-cause dementia risk in an MCI population. The brier score is used to assess the accuracy of probabilistic predictions over time, and ranges from 0 to 1, with a score closer to 0 indicating greater accuracy. Estimates were derived using bootstrapping. Biomarker values were natural log-transformed and standardized as Z scores. Aβ42/40 = Amyloid β42/40. GFAP = glial fibrillary acidic protein. MMSE = mini-mental state examination. NfL = neurofilament light. pTau181= phosphorylated-tau-181

**eFigure 2: Kaplan-Meier curves of clinical progression to AD dementia for individuals with low, medium, or high baseline plasma biomarker levels in the MCI subset.**


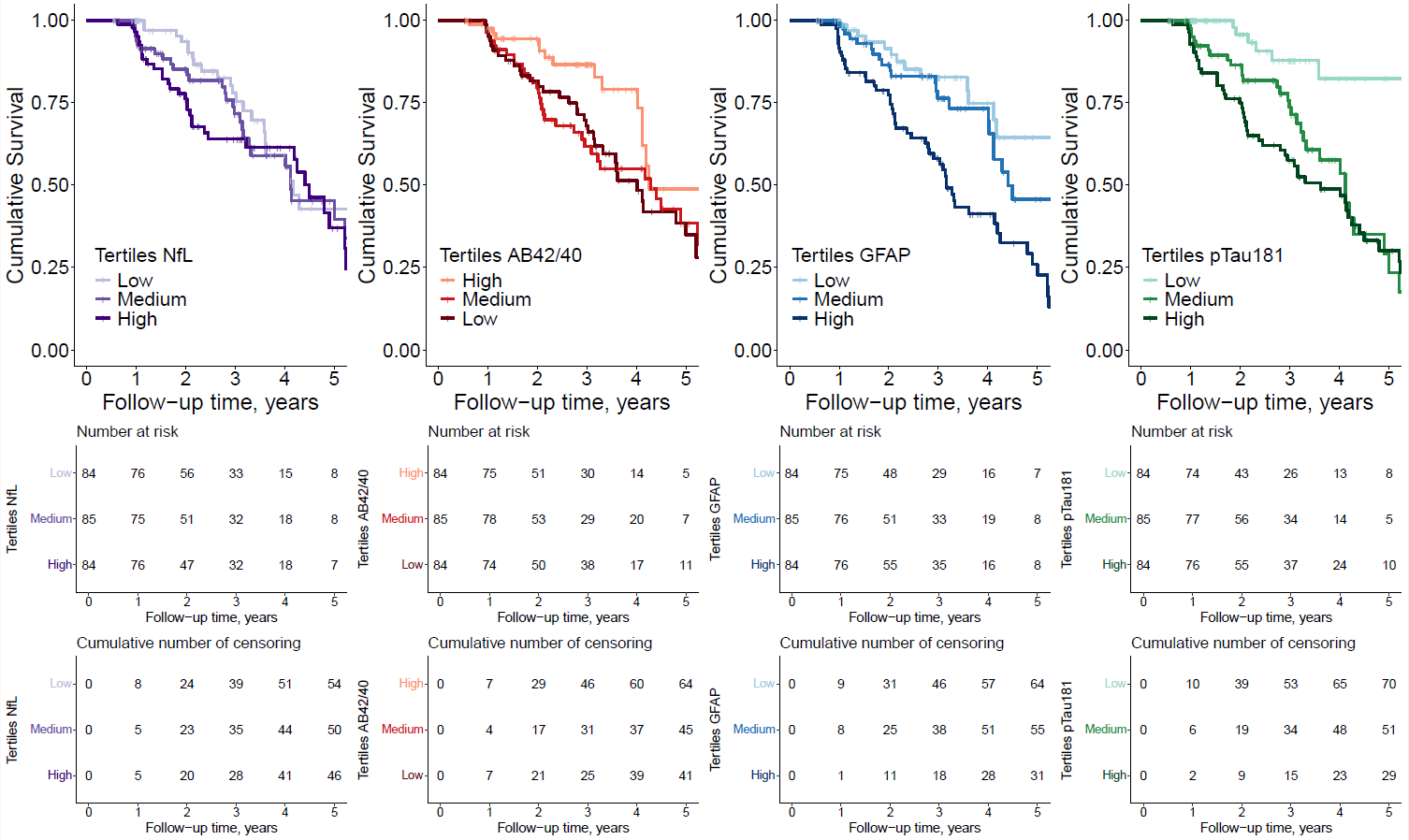


The Kaplan-Meier curves present the observed clinical progression to AD dementia over time, visualised for tertiles of baseline plasma biomarker concentrations. Curves are unadjusted for covariates age, sex and MMSE score. Aβ42/40 = Amyloid β42/40. GFAP = glial fibrillary acidic protein. NfL = neurofilament light. pTau181= phosphorylated-tau-181.

**eFigure 3: Brier scores of prognostic models over time for AD dementia as outcome.**


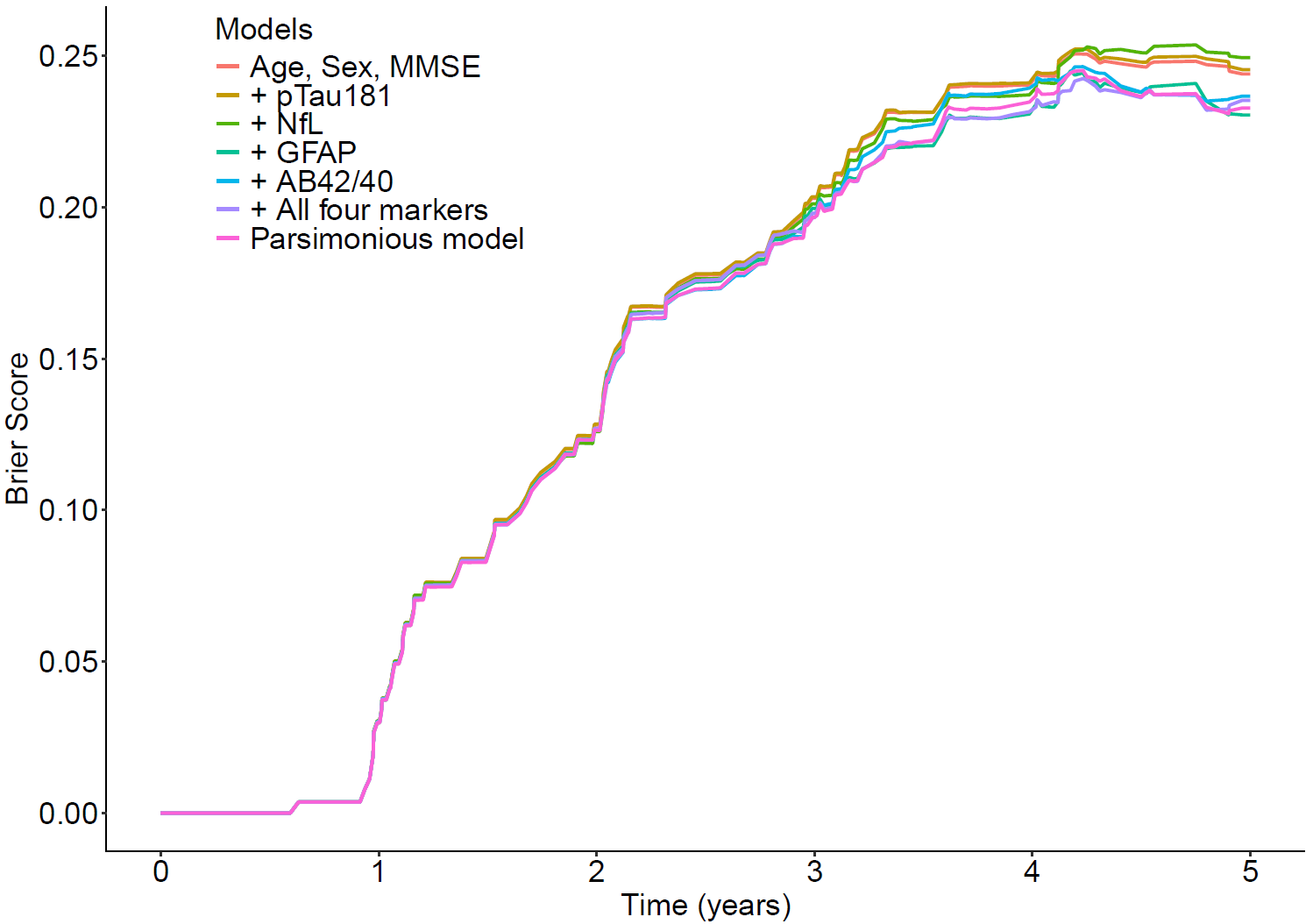


Evaluation of different cox regression prognostic models for AD dementia risk in an MCI population. The brier score is used to assess the accuracy of probabilistic predictions over time, and ranges from 0 to 1, with a score closer to 0 indicating greater accuracy. Estimates were derived using bootstrapping. Biomarker values were natural log-transformed and standardized as Z scores. The parsimonious model included age, sex, MMSE score, plasma GFAP and plasma pTau181. Aβ42/40 = Amyloid β42/40. GFAP = glial fibrillary acidic protein. MMSE = mini-mental state examination. NfL = neurofilament light. pTau181= phosphorylated-tau-181.

**eFigure 4: Brier scores of prognostic models over time for any cause dementia as outcome, including pTau217 data.**


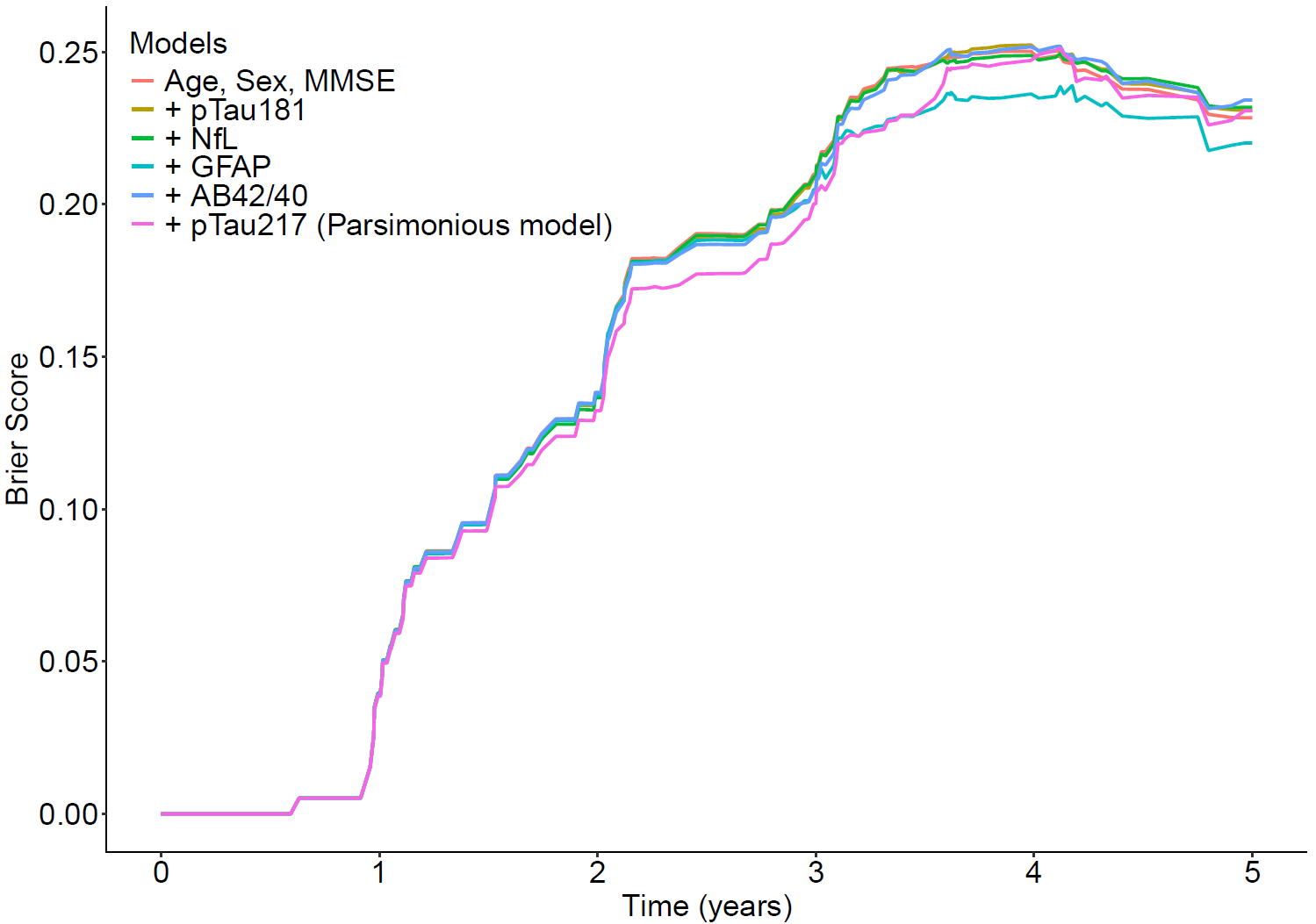


Evaluation of different cox regression prognostic models for any cause dementia risk in the MCI subset with pTau217 measurements. The brier score is used to assess the accuracy of probabilistic predictions over time, and ranges from 0 to 1, with a score closer to 0 indicating greater accuracy. Estimates were derived using bootstrapping. Biomarker values were natural log-transformed and standardized as Z scores. Aβ42/40 = Amyloid β42/40. GFAP = glial fibrillary acidic protein. MMSE = mini-mental state examination. NfL = neurofilament light. pTau181= phosphorylated-tau-181. pTau217= phosphorylated-tau-217.

**eFigure 5: Calibration of GFAP model performance for any-cause dementia as a clinical endpoint in ADC (left) MEMENTO (middle) and AIBL (right) cohorts.**

**
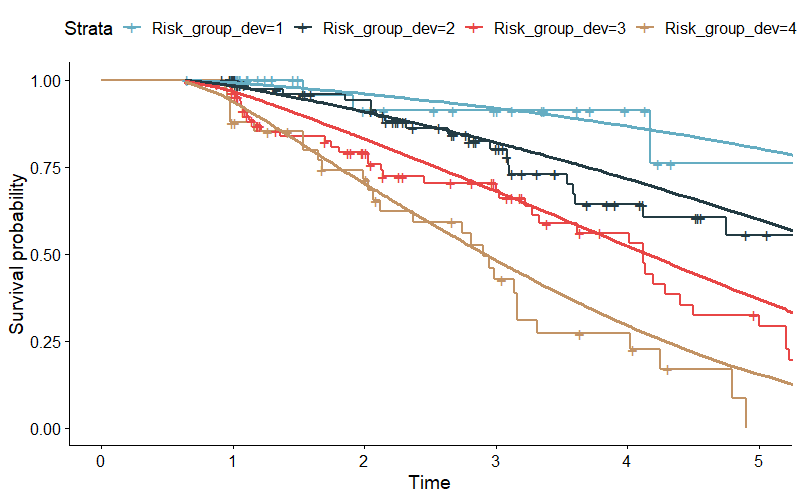

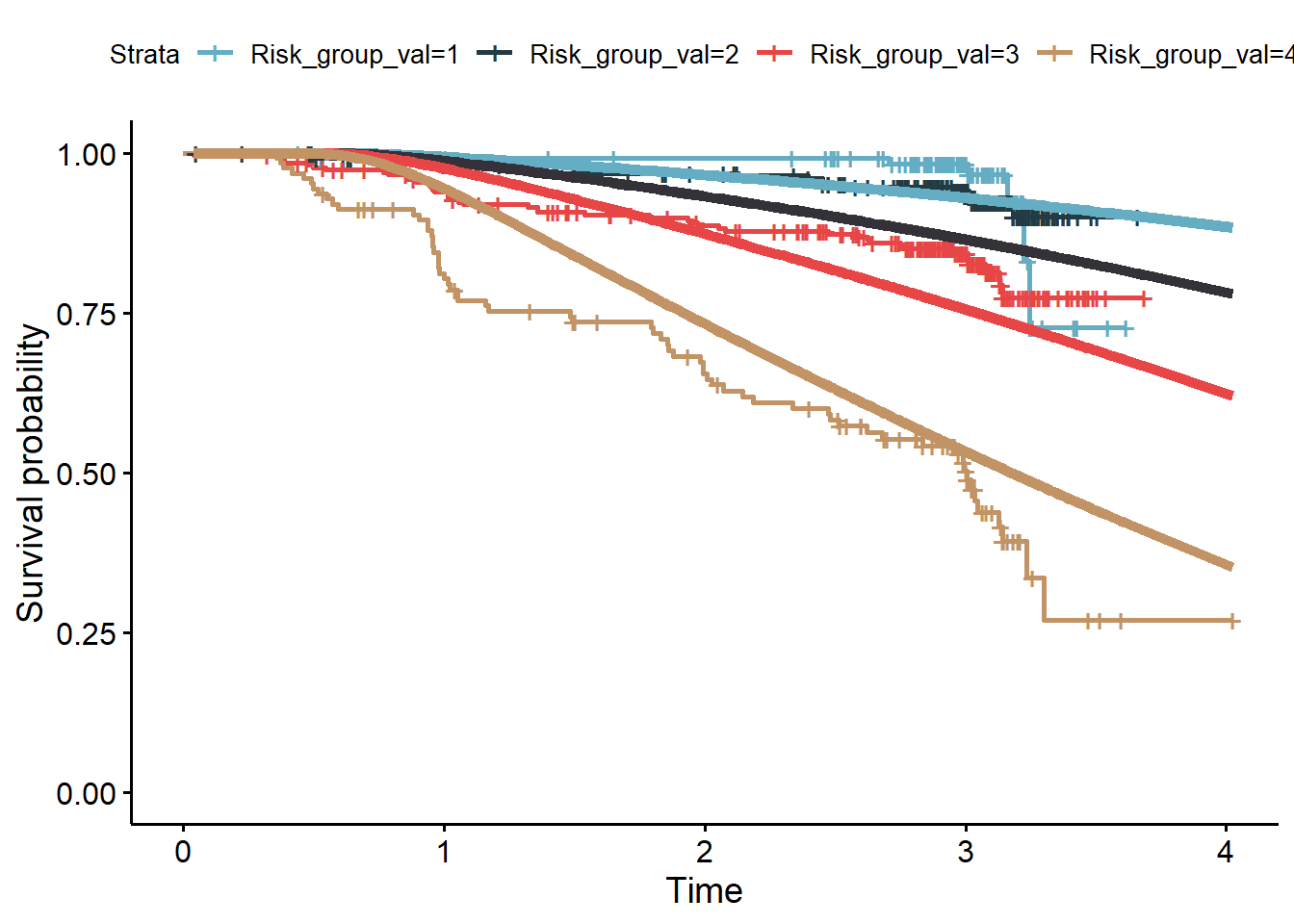

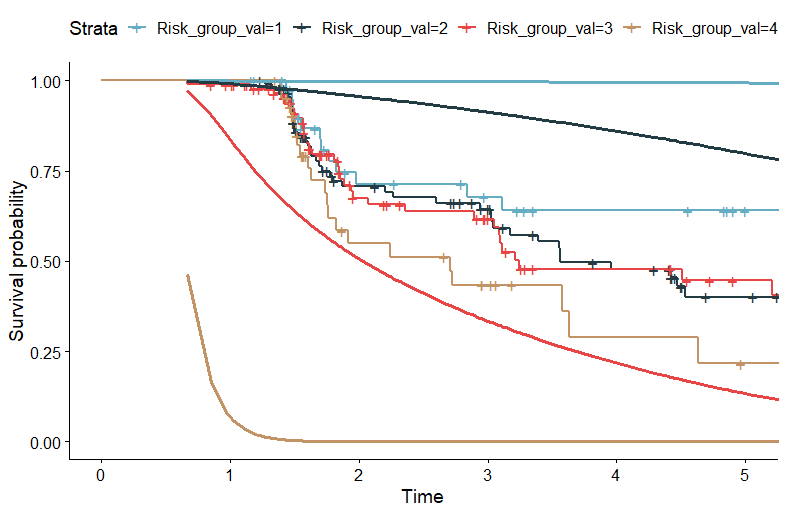
**

Smooth lines: predicted progression in the dataset analysed using the Cox model. Jagged lines: observed progression analysed by Kaplan-Meier. Risk groups were defined using the prognostic index of the model at the 16th, 50th and 84th centiles to obtain: good prognosis (>84th percentile, group 1), fairly good prognosis (50–84th percentile, group 2), fairly poor prognosis (16–50th percentile, group 3), and poor prognosis (<16th percentile, group 4).

**eFigure 6: Calibration of pTau217 model performance for any cause dementia as a clinical endpoint in ADC (left) and AIBL (right) cohorts.**

**
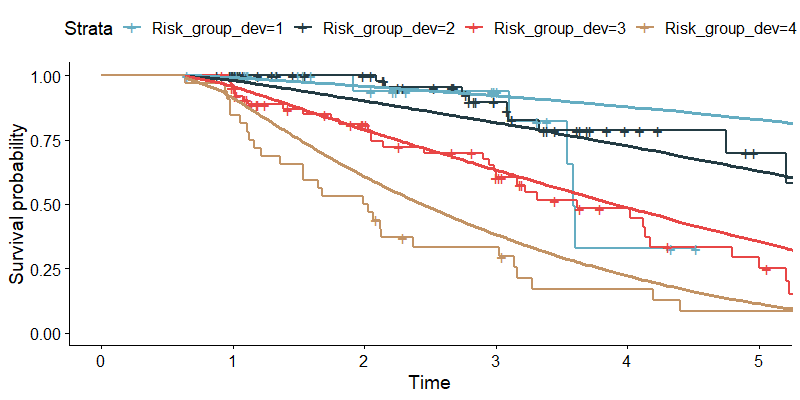

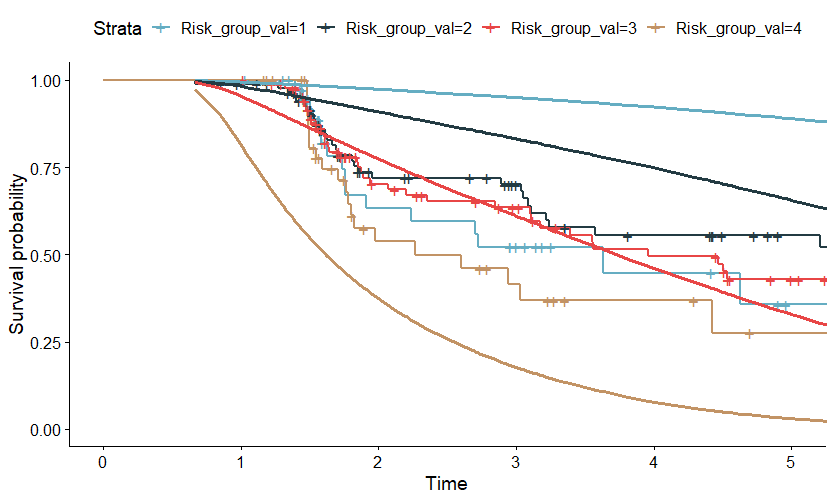
**

Smooth lines: predicted progression in the dataset analysed using the Cox model. Jagged lines: observed progression analysed by Kaplan-Meier. Risk groups were defined using the prognostic index of the model at the 16th, 50th and 84th centiles to obtain: good prognosis (>84th percentile, group 1), fairly good prognosis (50–84th percentile, group 2), fairly poor prognosis (16–50th percentile, group 3), and poor prognosis (<16th percentile, group 4).

**eFigure 7: Calibration of GFAP and pTau181 model performance for AD dementia as a clinical endpoint in ADC (left) MEMENTO (middle) and AIBL (right) cohorts**.


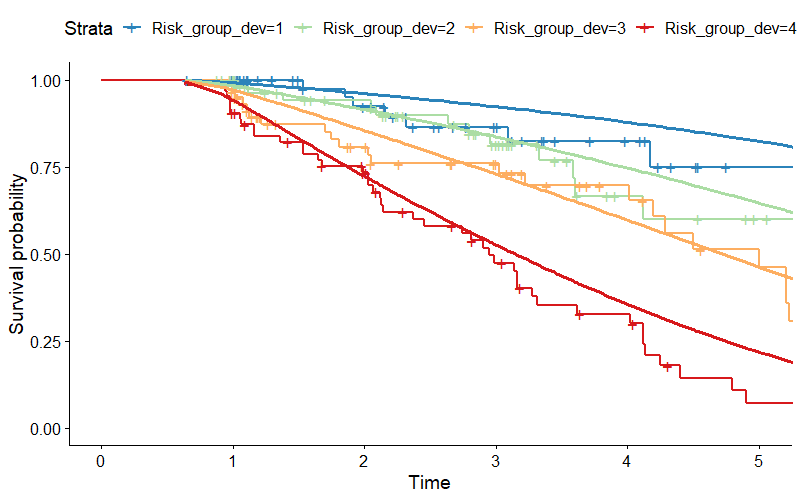

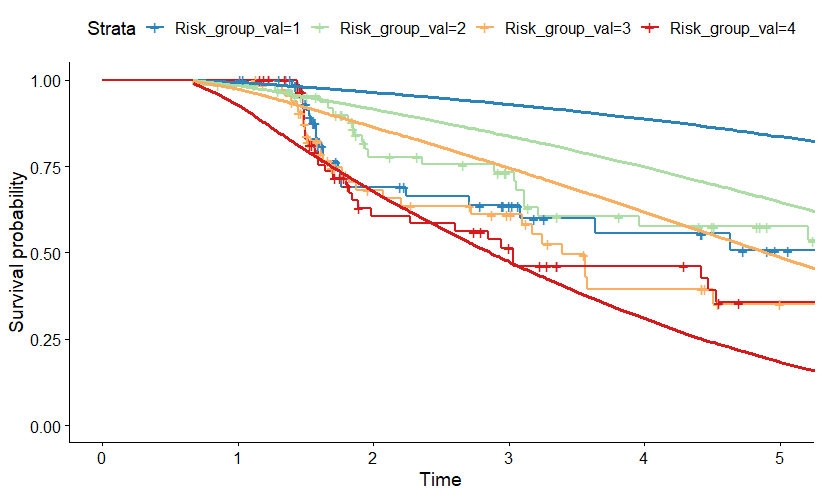

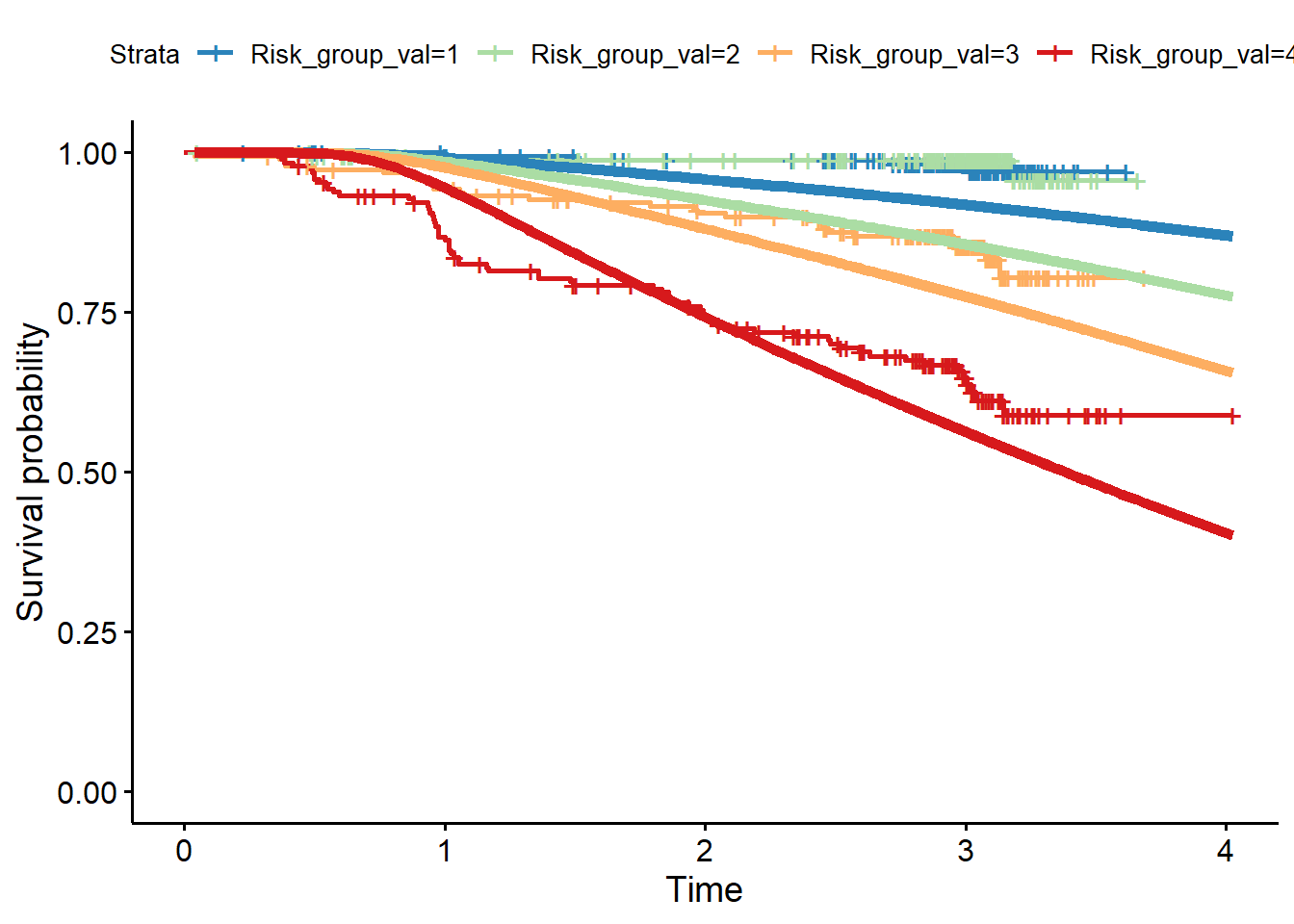


Smooth lines: predicted progression in the dataset analysed using the Cox model. Jagged lines: observed progression analysed by Kaplan-Meier. Risk groups were defined using the prognostic index of the model at the 25th, 50th and 75th centiles to obtain: good prognosis (>75th percentile, group 1), fairly good prognosis (50–75th percentile, group 2), fairly poor prognosis (25–50th percentile, group 3), and poor prognosis (<25th percentile, group 4).
